# The chromatin remodeler RSF1 controls centromeric histone modifications to coordinate chromosome segregation

**DOI:** 10.1038/s41467-018-06377-w

**Published:** 2018-09-21

**Authors:** Ho-Soo Lee, Zhonghui Lin, Sunyoung Chae, Young-Suk Yoo, Byung-Gyu Kim, Youngsoo Lee, Jared L. Johnson, You-Sun Kim, Lewis C. Cantley, Chang-Woo Lee, Hongtao Yu, Hyeseong Cho

**Affiliations:** 1 Department of Biochemistry, Ajou University School of Medicine, Suwon, 16499 Korea; 2 Genomic Instability Research Center, Ajou University School of Medicine, Suwon, 16499 Korea; 3 Department of Pharmacology, University of Texas Southwestern Medical Center, 6001 Forest Park Road, Dallas, TX 75930 USA; 4 Institute of Medical Science, Ajou University School of Medicine, Suwon, 16499 Korea; 5 Center for Genomic Integrity, Institute for Basic Science, UNIST, Ulsan, 44919 Korea; 6 Meyer Cancer Center, Department of Medicine, Weill Cornell Medical College, New York, NY 10065 USA; 7 Department of Molecular Biology, Sungkyunkwan University School of Medicine, Suwon, 16419 Korea; 8 Present Address: College of Chemistry, Fuzhou University, 350116 Fujian, China

## Abstract

Chromatin remodelers regulate the nucleosome barrier during transcription, DNA replication, and DNA repair. The chromatin remodeler RSF1 is enriched at mitotic centromeres, but the functional consequences of this enrichment are not completely understood. Shugoshin (Sgo1) protects centromeric cohesion during mitosis and requires BuB1-dependent histone H2A phosphorylation (H2A-pT120) for localization. Loss of Sgo1 at centromeres causes chromosome missegregation. Here, we show that RSF1 regulates Sgo1 localization to centromeres through coordinating a crosstalk between histone acetylation and phosphorylation. RSF1 interacts with and recruits HDAC1 to centromeres, where it counteracts TIP60-mediated acetylation of H2A at K118. This deacetylation is required for the accumulation of H2A-pT120 and Sgo1 deposition, as H2A-K118 acetylation suppresses H2A-T120 phosphorylation by Bub1. Centromeric tethering of HDAC1 prevents premature chromatid separation in RSF1 knockout cells. Our results indicate that RSF1 regulates the dynamics of H2A histone modifications at mitotic centromeres and contributes to the maintenance of chromosome stability.

## Abstract (web summary)

The chromatin remodeler RSF1 is enriched at mitotic centromeres but its function there is poorly understood. Here, the authors show that RSF1 regulates H2A phosphorylation and acetylation at mitotic centromeres and contributes to chromosome stability.

## Introduction

The regulation of chromatin structure is essential for the preservation of genome integrity. ATP-dependent chromatin remodeling complexes control nucleosome movement and repositioning during DNA replication and DNA repair and other chromatin-templated processes^1,2^. The remodeling and spacing factor (RSF) belongs to the ISWI family of chromatin remodeling complexes and is composed of RSF1 and the ATPase SNF2H^3,4^. RSF1 localizes to DNA lesions and promotes efficient DNA repair by both homologous recombination and non-homologous end joining (NHEJ)^5–7^. RSF1 is also enriched at interphase centromeres^8,9^, and regulates NHEJ by recruiting the CENP-S–CENP-X centromere proteins to sites of DNA damage^5,7^. In addition, RSF1 depletion leads to chromosome aberrations in mitosis^9,10^. The mitotic functions of RSF1 have begun to be understood. For example, we have recently shown that RSF1 localizes to mitotic centromeres and recruits PLK1 for stable kinetochore-microtubule attachment^11^. It remains possible, however, that RSF1 plays other functions at mitotic centromeres.

Human sister chromatids at metaphase are primarily linked by cohesion ring complex at centromeres showing iconic X shape^12,13^. Centromeres are specialized chromatin composed of highly repetitive α-satellite DNA in humans^14^ and functional centromeres are marked by the presence of the centromere-specific histone H3-variant, CENP-A^15,16^. The primary function of the centromere is to provide the foundation for kinetochore assembly^17^, and the kinetochore provides the attachment site for microtubules and spindle checkpoint protein complexes^18–20^. During prophase of human cells, cohesin from chromosome arms is displaced in a non-proteolytic manner. Mitotic kinases phosphorylate cohesin and its positive regulator sororin, and these phosphorylation events open the cohesion ring complex and trigger the release of cohesin from the chromosome arms^21–23^. At centromeres, sororin and cohesion are protected from phosphorylation by the shugoshin1 (Sgo1) and protein phosphatase 2A (PP2A) complex^24,25^. The Sgo1–PP2A complex dephosphorylates cohesin and sororin^23–25^, preserving centromeric cohesion until the metaphase–anaphase transition. Sgo1 is recruited to kinetochores through binding to phosphorylated histone H2A on Thr120 (H2A-pT120), which is generated by the kinetochore kinase, Bub1^26,27^. The localization of Sgo1 to inner centromeres and kinetochores is further regulated by microtubule-kinetochore tension and mitotic transcription at centromeres^28,29^. The maintenance of centromeric cohesion is crucial for preventing premature chromosome segregation and chromosome instability^27^.

In the present study, we show that premature chromosome segregation in RSF1 knockout (KO) cells is triggered by defects in Sgo1 binding to centromeres. We further show that RSF1 recruits HDAC1 to centromeres, and HDAC1-mediated deacetylation of H2A at K118 is a prerequisite for the accumulation of H2A-pT120 and Sgo1 deposition. We propose that RSF1 coordinates a crosstalk between histone acetylation and phosphorylation and tunes histone marks at centromeres to maintain chromosome stability.

## Results

### RSF1 is required for the protection of centromeric cohesion

**Fig. 1:**
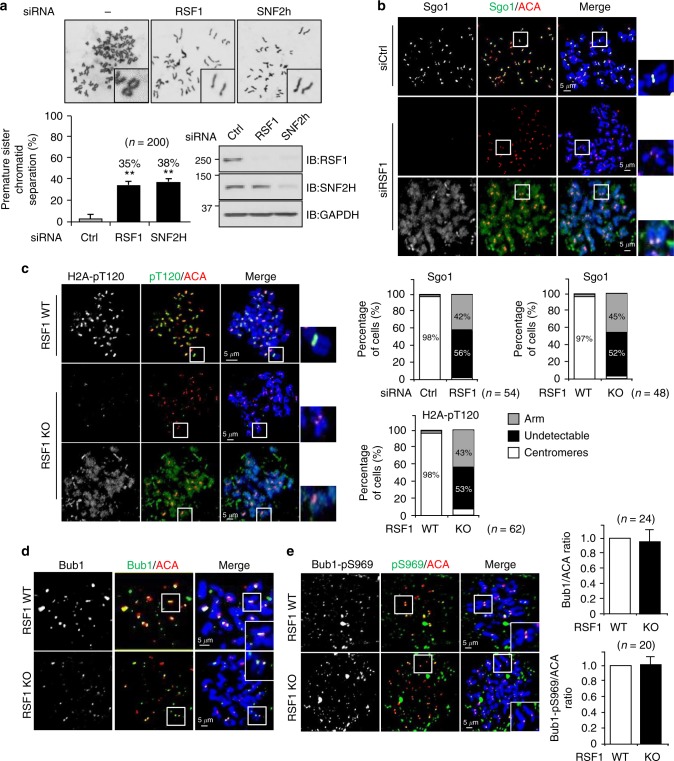
RSF1 is necessary for restricting H2A-pT120 and Sgo1 to centromeres. **a** HeLa cells were transfected with siRNAs, and floating mitotic cells were obtained after nocodazole treatment for 4 h and subjected to metaphase chromosome spread stained with Giemsa. Quantification of sister chromatid separation in HeLa cells after depletion of RSF1 or SNF2H. Data represent mean ± SEM; **p < 0.01 vs. control siRNA by Student’s *t*-test. **b** HeLa cells were transfected with siRNAs, and RSF1-depleted cells were subjected to chromosome spread immunostaining. Images were obtained from representative mitotic cells: Sgo1 (green), ACA (red), and DAPI (blue). The percentages of cells exhibiting the arm or centromeric localizations of Sgo1 proteins are shown. Scale bar, 5 μm. **c** RSF1 knockout mitotic cells were analyzed by immunofluorescence staining: H2A-pT120 (green), ACA (red), and DAPI (blue). Percentage of cells exhibiting the arm or centromeric expression level of H2A-pT120 are shown. Scale bar, 5 μm. **d** Localization of Bub1 on mitotic chromosomes in RSF1 WT or knockout (KO) cells. Nocodazole-treated, mitotic RSF1 WT or knockout (KO) HeLa cells were stained with DAPI and the Bub1 antibodies. The graph represents relative intensity of Bub1 against ACA at kinetochores. At least 100 kinetochores of prometaphase cells were analyzed in three independent experiments. Scale bar, 5 μm. **e** Metaphase chromosome spreads were stained with anti-Bub1-pS969 and anti-ACA in RSF1 WT or KO cells. Cells were stained with DAPI and the Bub1-pS969-specific antibodies. The graph represents relative intensity of Bub1-pS969 against ACA at kinetochores. At least 100 kinetochores of prometaphase cells were analyzed in three independent experiments. Scale bar, 5 μm

We recently showed that the chromatin remodeler RSF1 is enriched at mitotic centromeres and contributes to the recruitment of PLK1 to centromeres^11^. RSF1-deficient cells exhibited chromosome aberrations^9,10^, suggesting that centromeric cohesion might be aberrantly regulated. To test this hypothesis, we performed metaphase chromosome spreads of RSF1 RNAi cells. RSF1 knockdown (KD) resulted in premature sister chromatid separation (PSCS) with loss of primary constriction (Fig. 1a). Similar results were obtained in cells with SNF2H KD, which also resulted in a significantly reduced RSF1 protein level^11^. Reconstitution of RSF1-V5 in RSF1 KO cells partially rescued the PSCS phenotype (Supplementary Fig. 1a).

Protection of centromeric cohesion is crucial for preventing premature chromosome segregation, and centromeric localization of Sgo1 protects against premature cohesin removal^23^. Sgo1 was co-stained with anti-centromere antibody (ACA) as an inner kinetochore marker in HeLa cells. siRNA-mediated RSF1 depletion or KO severely impaired the localization of Sgo1 to centromeres. As a result, Sgo1 was distributed along the chromosome arm (in ~40% of the cells) or was undetectable (Fig. 1b and Supplementary Fig. 1b). Loss of Sgo1 protein in RSF1 KO cells is not due to changes in its protein level. In direct immunofluorescence staining, diffused pattern of Sgo1 in RSF1 KO cells is detected in the cytosol (Supplementary Fig. 1c, d). Centromeric localization of Sgo1 requires histone H2A phosphorylation at T120 (H2A-pT120) by the kinase Bub1^26–29^. Although H2A-pT120 was confined to centromeres in HeLa cells, it showed diffuse staining throughout the chromosome arms (43%) or was undetectable in RSF1 KO cells (Fig. 1c) that were previously established in our lab^11^.

By contrast, centromeric localization of Bub1 (Fig. 1d) and the autophosphorylation of Bub1 were not affected by the absence of RSF1 (Fig. 1e). This was verified by Western blot analysis of the chromatin fraction (Supplementary Fig. 1e). The autophosphorylation of Bub1 was not affected by the presence or absence of RSF1. RSF1 KO increased the accumulation of Sgo1 on chromatin, whereas re-expression of RSF1 decreased it, indicating that loss of centromeric Sgo1 increases the chromatin distribution of Sgo1 along the chromosome arm. Similarly, RSF1 KO increased H2A-pT120 in the chromatin fraction, whereas re-expression of RSF1 decreased H2A-pT120 levels (Supplementary Fig. 1e). Taken together, our data demonstrate that RSF1 is necessary for restricting H2A-pT120 and Sgo1 to centromeres and for the protection of centromeric cohesion.

### Acetylation of H2A-K118 suppresses H2A-T120 phosphorylation

**Fig. 2:**
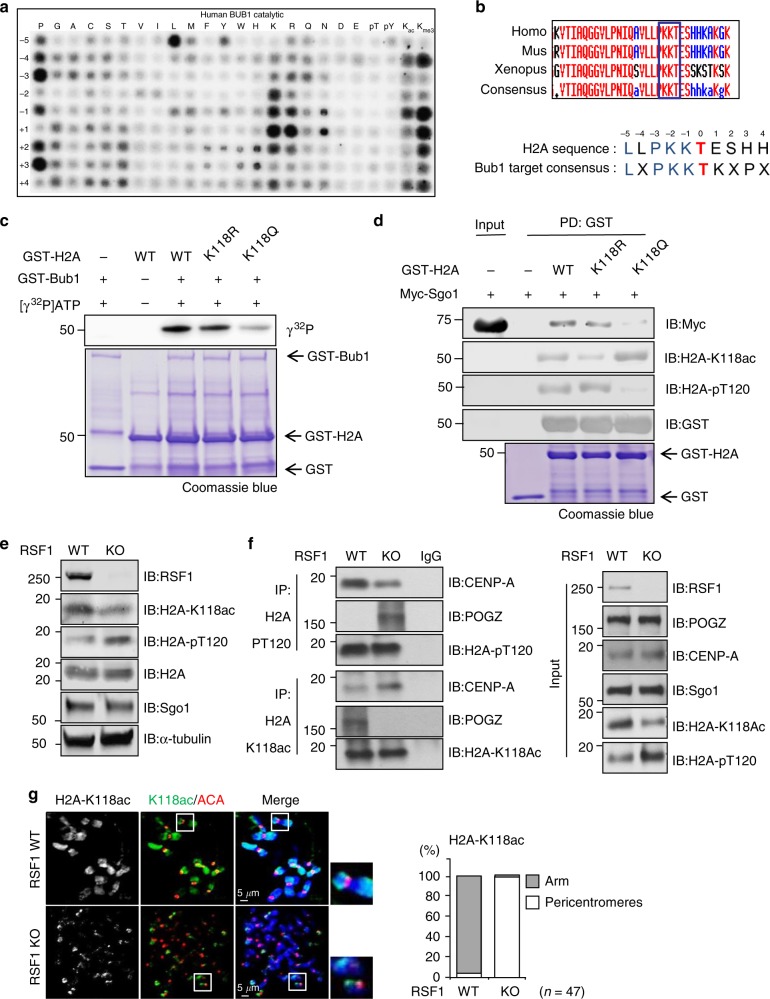
Acetylation of H2A-K118 suppresses H2A-T120 phosphorylation and Sgo1 deposition. **a** Individual in vitro kinase assays were carried out with recombinant GST-Bub1 kinase and a positional scanning peptide library consisting of 189 biotinylated substrate peptides. **b** Alignment of vertebrate H2A sequence with the conserved Bub1 consensus motif. **c** In vitro kinase assay: recombinant GST-H2A proteins purified from bacteria were incubated with the GST-Bub1 at 30 °C for 30 min in the presence of γ^32^P-ATP. Incorporation of ^32^P into H2A protein was visualized by autoradiography. Coomassie blue staining demonstrated equal protein loading. **d** Recombinant GST-H2A proteins were incubated with Myc-tagged Sgo1 expressing mitotic whole cell lysates for 12 h at 4 °C and subjected to immunoblotting. **e** RSF1 WT or KO HeLa cells were treated with paclitaxel for 16 h. Mitotic cell lysates were analyzed by immunoblotting. **f** Mitotic RSF1 WT or KO HeLa cell lysates were immunoprecipitated using anti-H2A-pT120 or anti-H2A-K118ac antibodies. The inputs and immunoprecipitates were analyzed by western blot using anti-CENP-A or anti-POGZ. CENP-A or POGZ proteins are specifically localized on the mitotic centromeres and chromosome arms, respectively. **g** Mitotic HeLa cells were stained with DAPI and H2A-K118ac (green), ACA (red), and DAPI (blue) antibodies. The percentages of cells exhibiting the chromosome arm or centromeric localizations of H2A-K118 acetylation levels are shown. Scale bar, 5 μm

Because Bub1 localization and kinase activity were intact in RSF1 KO cells, we hypothesized that Bub1-mediated phosphorylation of H2A might be inhibited by neighboring post-translational modifications. We performed an in vitro kinase assay with the recombinant Bub1 kinase domain as the enzyme and a peptide library array as substrates (Fig. 2a). The H2A substrate peptide [LLPKK(T)ESHH] of Bub1 (Fig. 2b) is used as a starting peptide. Each amino acid positioned from −5 to +4 was switched from P (Pro) to Kme3 indicated at the Y-axis. The assay revealed Bub1 strongly prefers a leucine residue at the −5 position. Several major Bub1 substrates, including H2A-pT120 and CDC20 (S153), all contain a leucine at this position^29,30^. Bub1 also prefers a lysine residue at the −2 position (Fig. 2a). Interestingly, substitution of this lysine to acetyllysine (K_ac_) or trimethylated lysine (K_me3_) greatly reduced Bub1 phosphorylation. This result suggests that modification of the −2 lysine might inhibit Bub1 phosphorylation.

In H2A, two upstream lysine residues of K118 and K119 are well conserved among species (Fig. 2b). The mass spectrum analysis has uncovered various histone modifications. It showed acetylation of H2A K118 but its biological function is not addressed^31,32^. To test whether acetylation of K118 affected H2A-T120 phosphorylation by Bub1, we replaced the Lys 118 residue of H2A to Arg (H2A-K118R). In addition, an acetylation mimicking mutant of H2A-K118Q (Lys118 to Gln) was generated. The GST-tagged H2A variants were purified in bacteria and subjected to in vitro kinase assay. As shown in Fig. 2c, phosphorylation on H2A-K118Q by Bub1 kinase was significantly reduced, whereas phosphorylation of the H2A-K118R variant was similar to that of wild type H2A, suggesting that acetylation of the Lys118 inhibits the phosphorylation of H2A by Bub1. Next, the immobilized histone variants were incubated with mitotic cell lysates expressing Myc-Sgo1 and analyzed by immunoblotting for analysis of histone modifications and interactions. As expected, H2A-K118Q displayed a strong H2A-K118 acetylation with a weak H2A-T120 phosphorylation. And Sgo1 binding to the H2A-K118Q was also substantially reduced (Fig. 2d). We verified that immobilized H2A histone variants were tightly associated with other histones such as H2B, H3, and H4 (Supplementary Fig. 2a), indicating that GST-H2A was incorporated into nucleosomes in these experiments, which allows Sgo1 binding^29^. By contrast, H2A-pT120 phosphorylation of H2A-K119Q remained unchanged and Sgo1 binding to H2A-K119R and H2A-K119Q were as efficient as to wild type H2A (Supplementary Fig. 2b). Together, these findings indicated that H2A-pT120 phosphorylation and Sgo1 binding were modulated by the acetylation of neighboring H2A-K118, but not of H2A-K119.

Next, we investigated whether there was an inverse relationship between H2A-pT120 and H2A-K118ac in mitotic HeLa cells. Immunoblotting of whole cell lysates (WCL) showed that, in RSF1-expressing HeLa cells, the level of H2A-K118ac was high and that of H2A-pT120 was relatively low, whereas this inverse relationship was reversed in RSF1 KO cells (Fig. 2e). Because maintenance of H2A-pT120 at centromeres is crucial for proper chromosome segregation, we designed an experiment to distinguish the modifications of H2A at centromeres from those at chromosome arms. We used CENP-A^19^ as a centromere marker and POGZ as a marker for chromosome arms^33^, and the expression of these proteins was confirmed by immunofluorescence staining (Supplementary Fig. 2c). RSF1 depletion did not affect the expression levels of CENP-A and POGZ, and co-immunoprecipitation verified that they were independent from each other (Supplementary Fig. 2d). To determine the distribution of H2A-pT120 on the chromatin), we immunoprecipitated H2A-pT120 in mitotic cell lysates and found that a subset of CENP-A, but not of POGZ, exists in a complex with H2A-pT120 (Fig. 2f), indicating that H2A-pT120 localizes to centromeres. In RSF1 KO cells, H2A-pT120 co-precipitated with both CENP-A and POGZ, and CENP-A in the H2A-pT120 immunoprecipitate was reduced. These results are consistent with the findings in Fig. 1c, which demonstrated the reduction of H2A-pT120 at centromeres and the spread of this mark to chromosome arms. On the other hand, a major portion of H2A-K118ac was found to form a complex with POGZ in mitotic cells. In RSF1 KO cells, H2A-K118ac only pulled down CENP-A and not POGZ. Therefore, centromeric accumulation of H2A-K118ac was accompanied by loss of H2A-pT120 at centromeres in RSF1 KO cells. We next examined the chromosome localization of H2A-K118ac in mitotic cells by immunofluorescence staining. H2A-K118ac in HeLa cells was detected throughout the chromosome arms, whereas it was confined to the centromeric region neighboring ACA in RSF1 KO cells (Fig. 2g). Double-immunostaining with H3K9me3 as a pericentromeric marker revealed that H2AK118ac is enriched at pericentromeres of RSF1-KO cells (Supplementary Fig. 2e). Our findings suggest that the histone marks at centromeres are regulated by the RSF1 chromatin remodeler and that Bub1-mediated H2A-T120 phosphorylation at centromeres is antagonized by the acetylation of H2A-K118.

### RSF1 recruits HDAC1 to centromeres to regulate histone H2A acetylation

**Fig. 3:**
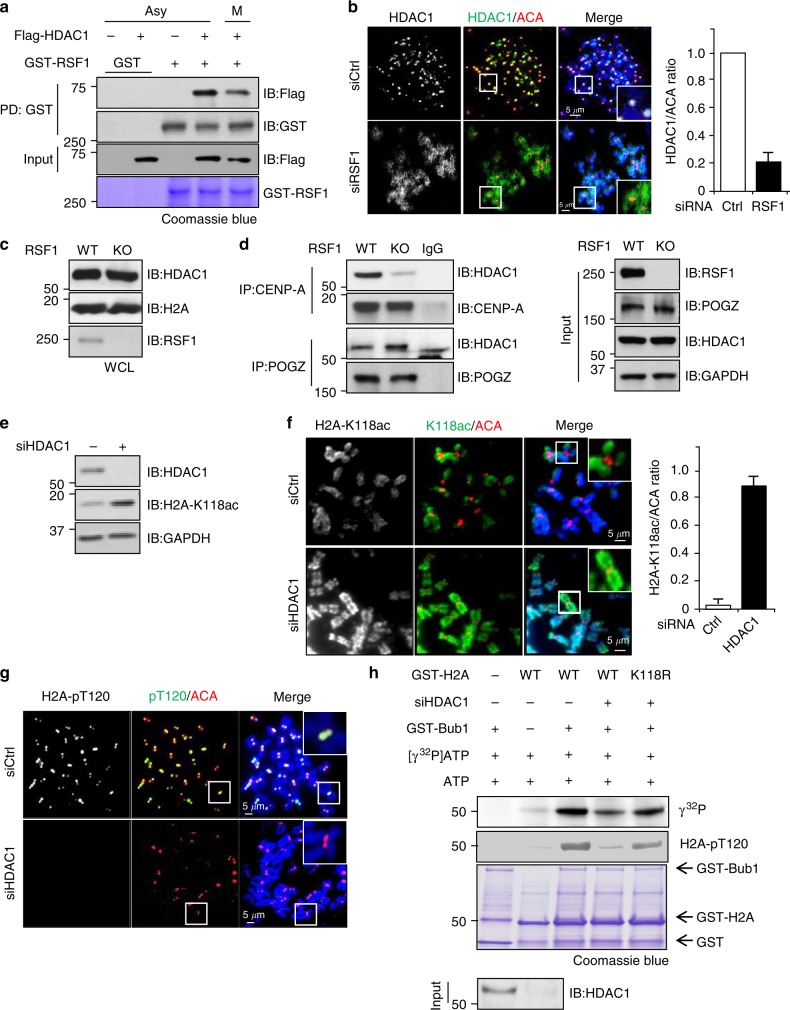
RSF1 recruits HDAC1 to centromeres and regulate histone H2A deacetylation. **a** Recombinant GST-RSF1 was incubated with Flag-HDAC1 expressing asynchronously growing cells (Asy) or mitotic cells (M) for 2 h at 4 °C. GST-RSF1 bound to Flag-HDAC1 was detected by immunoblotting. Recombinant GST-RSF1 was stained with Coomassie blue. **b** HeLa cells were transfected with control or RSF1 siRNA, and floating mitotic cells were obtained after nocodazole treatment for 4 h and subjected to chromosome spread immunostaining. Metaphase chromosome spreads were stained with anti-HDAC1 (green), anti-ACA (red), and DAPI (blue). The graph represents relative intensity of HDAC1 against ACA at kinetochores. Prometaphase cells were analyzed in three independent experiments. Scale bar, 5 μm. **c** RSF1 WT or RSF1-knockout (KO) mitotic HeLa cell lysates were blotted with the indicated antibodies. **d** Endogenous CENP-A (centromere protein) or POGZ (chromosome arm protein) were immunoprecipitated from nocodazole arrested RSF1 WT or RSF1 knockout HeLa cells and blotted with the indicated antibodies. **e** Immunoblot analysis of histone H2A acetylation levels in mitotic HeLa cells. Cells were transfected with control or HDAC1 siRNA for 48 h and floating mitotic cells were analyzed by immunoblot. **f** HeLa cells were transfected with control or HDAC1 siRNA, and floating mitotic cells were subjected to chromosome spread immunostaining and stained with anti-H2A-K118ac and ACA antibodies. Scale bar, 5 μm. **g** HeLa cells were transfected with control or HDAC1 siRNA, and floating mitotic cells were subjected to chromosome spread immunostaining and stained with anti-H2A-pT120 and ACA antibodies. Scale bar, 5 μm. **h** Recombinant GST-H2A proteins were incubated with control or HDAC1 siRNA transfected HeLa cells. GST-H2A proteins were incubated with recombinant GST-Bub1 in the presence of γ^32^P-ATP. Incorporation of ^32^P into H2A protein was visualized by autoradiography

Because RSF1 does not catalyze protein modifications, it likely exerts its effects through interacting proteins. We thus screened for RSF1-interacting proteins using tandem affinity purification followed by mass spectrometry analysis, which indicated HDAC1 as a potential RSF1-binding protein (Supplementary Data 1). Although the transcriptional function of HDAC1 has been studied extensively^34–36^, its subcellular localization and function in mitosis was partly shown^37,38^. Flag-HDAC1 bound to GST-RSF1 in human cell lysates, and the interaction between these proteins was maintained in asynchronously growing cells (mainly interphase) and mitotic cells (Fig. 3a). Immunofluorescence staining showed that HDAC1 was enriched at mitotic centromeres and co-stained with ACA. RSF1 KD markedly increased the distribution of HDAC1 throughout the chromosome arms (Fig. 3b). This was accompanied with an increase of chromatin-bound HDAC1 levels whereas HDAC2 and HDAC3 remained unchanged in these cells (Supplementary Fig. 3a). However, centromeric HDAC1 in these cells was dramatically decreased (Fig. 3b, right panel), suggesting that the centromeric tethering of HDAC1 was regulated by binding to RSF1. The total amount of HDAC1 in WCL did not change in RSF1 KO cells (Fig. 3c). Consistent with the staining data, the amount of HDAC1 co-precipitated by anti-CENP-A antibody was greatly reduced in RSF1 KO cells, whereas the association of HDAC1 with POGZ increased in RSF1 KO cells (Fig. 3d). These data demonstrate that RSF1 tethers HDAC1 to centromeres.

Next, we addressed whether HDAC1 regulated histone H2A-K118 acetylation. Depletion of HDAC1 increased H2A-K118ac in mitotic WCL (Fig. 3e). Consistently, H2A-K118ac levels were elevated throughout the chromosome in HDAC1-depleted cells, including the centromeric region, which showed high levels of H2A-K118ac (Fig. 3f). In these cells, the levels of centromeric H2A-pT120 (Fig. 3g) and Sgo1 (Supplementary Fig. 3b) were dramatically decreased, indicating that HDAC1 is necessary for the phosphorylation of H2A-T120 and Sgo1 deposition during mitosis. Consistent with these observations, in in vitro kinase assay, Bub1-mediated phosphorylation of H2A-T120 was suppressed in the absence of HDAC1 (Fig. 3h). On the other hand, phosphorylation of H2A-K118R in the absence of HDAC1 was nearly as effective as of H2A-WT in the presence of HDAC1 (Fig. 3h and Supplementary Fig. 3c). Similar results regarding H2A-T120 phosphorylation and Sgo1 deposition were obtained in cells treated with TSA (Trichostatin A), an HDAC inhibitor (Supplementary Fig. 3d, e). Taken together, these data indicate that the levels of H2A-pT120 and H2A-K118ac at centromeres are regulated by HDAC1.

### The RSF1-HDAC1 interaction is required for H2A-pT120 maintenance

**Fig. 4:**
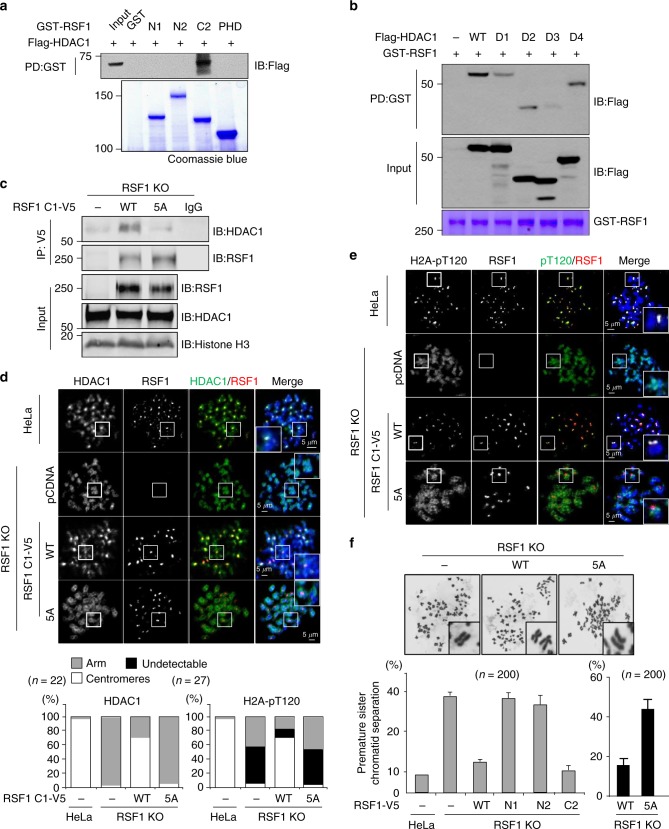
The RSF1-HDAC1 interaction is required for faithful chromosome segregation. **a** Recombinant GST-RSF1 proteins were incubated with Flag-HDAC1 expressing mitotic lysates and subjected to immunoblotting. N1: amino acids 1–627, N2: 1–871, C2: 982–1441, PHD (plant homeodomain): 628–973. **b** GST-RSF1 was incubated with Flag-HDAC1 deletion mutants expressing mitotic cell lysates for 1 h at 4 °C. Precipitates were subjected to immunoblotting with anti-Flag. D1: amino acids 50–482, D2: 250–482, D3: 1–325, D4: 1-430. **c** The RSF1-C1 WT or 5A mutant was transfected into RSF1 KO cells, and mitotic lysates were immunoprecipitated with anti-V5 antibody, followed by immunoblotting. **d**, **e** RSF1 WT or KO HeLa cells were introduced with RSF1-C1 WT or 5A mutant and treated with nocodazole for 4 h. Floating mitotic cells were subjected to chromosome spread immunostaining with anti-HDAC1 or anti-H2A-pT120 and anti-RSF1 antibodies. The percentages of cells exhibiting the arm or centromeric localizations of HDAC1 proteins or H2A-pT120 level were plotted on a graph. **f** HeLa cells transfected with RSF1 WT or indicated mutants in RSF1 KO cells were subjected to metaphase chromosome spread and stained with Giemsa. Quantification of the percentage of premature sister chromatid separation in HeLa cells were shown. Scale bar, 5 μm

Because RSF1 binds HDAC1, we next mapped their binding domains using deletion mutants. PD assays identified the C-terminal region (C2: aa 982–1441) of RSF1 as the HDAC1-binding region (Fig. 4a and Supplementary Fig. 4a). Likewise, the C-terminal region of HDAC1 (Fig. 4b and Supplementary Fig. 4b) was required for its interaction with RSF1. We searched for any putative binding motifs in the C-terminal region of RSF1 and found that LSSSE as an LXCXE-like motif^37^ is conserved among higher vertebrates (Supplementary Fig. 4c). We generated an RSF1-5A mutant in which the five amino acids in the C-terminal LXCXE motif were mutated to alanine (5A). Co-immunoprecipitation experiments showed that the RSF1-5A mutant lost most of its binding ability to HDAC1 (Fig. 4c). Accordingly, centromeric HDAC1 was restored in RSF1 KO cells expressing the C-terminal region (C1) of RSF1 WT, but not that of RSF1 5A (Fig. 4d). Similarly, centromeric H2A-pT120 was recovered in RSF1 KO cells by the expression of RSF1 C1-WT, but not RSF1 C1-5A (Fig. 4e).

We next addressed whether the RSF1-HDAC1 interaction is necessary for proper chromosome segregation. PSCS observed in RSF1 KO cells (Fig. 1a) was prevented by the expression of RSF1-C2, but not N1 and N2 (Fig. 4f and Supplementary Fig. 4d). Expression of RSF1-5A did not prevent PSCS in RSF1 KO cells (Fig. 4f). The increase in the levels of chromatin-bound HDAC1 and Sgo1 in RSF1 KO cells was suppressed by RSF1-C2, but not by N1 and N2 (Supplementary Fig. 4e). These data demonstrate that the RSF1-HDAC1 interaction is crucial for the prevention of premature chromosome segregation through the modulation of H2A-pT120.

### Centromere-tethered HDAC1 bypasses the RSF1 function in mitotic cells

**Fig. 5:**
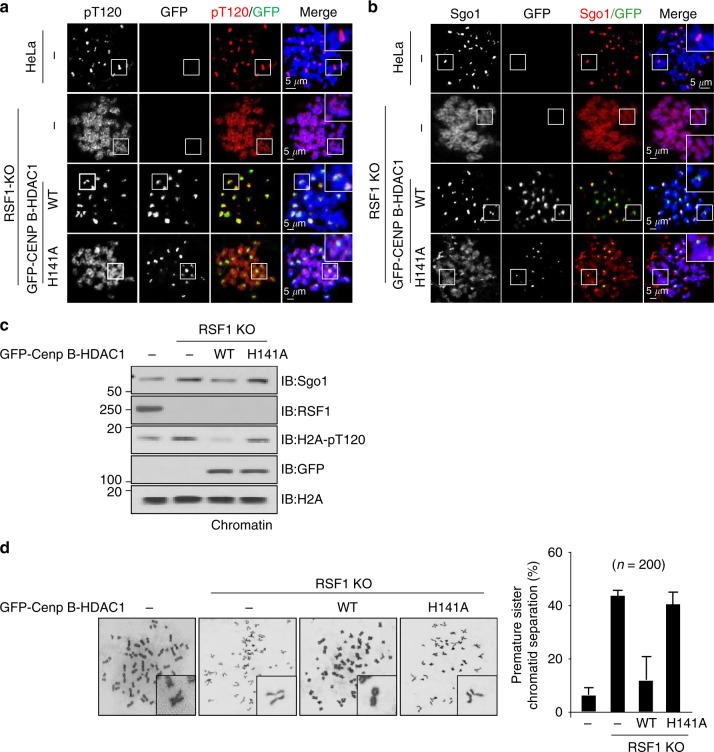
RSF1-deficient cells suffering from sister chromatid cohesion defects are rescued by centromere targeting HDAC1. **a** RSF1 KO HeLa cells were transfected with centromere targeting GFP-CENP B fused-HDAC1 WT or H141A catalytic dead mutant constructs. Floating mitotic cells were obtained after nocodazole treatment for 4 h and subjected to chromosome spread immunostaining with anti-H2A-pT120 (red) antibodies. Scale bar, 5 μm. **b** The cells prepared as Fig. 5a were subjected to chromosome spread immunostaining with anti-Sgo1 (red), and DAPI (blue). Scale bar, 5 μm. **c** Immunoblotting of chromatin fractions in RSF1 KO cells after reintroduction of CENP B fused GFP-HDAC1. **d** Giemsa staining of the metaphase chromosome spreads prepared after transfection of GFP-CENP B fused HDAC1 WT or H141A in RSF1 KO cells. Left panel shows quantification of the percentage of premature sister chromatid separation

If an important function of RSF1 in chromosome segregation is the recruitment of HDAC1 to mitotic centromeres, we would expect that centromere tethering of HDAC1 might restore Sgo1 deposition at centromeres in RSF1 KO cells. To test this hypothesis, centromere-tethered HDAC1 was generated by fusing it to CENP-B, whose DNA-binding domain binds the centromeric α-satellite DNA^39,40^. GFP was also fused at the N-terminus for visualization. As shown in Fig. 5a, centromeric H2A-pT120 was restored in RSF1 KO cells by the introduction of CENP-B-HDAC1, but not by the CENP-B-fused, catalytically inactive HDAC1 H141A mutant. Furthermore, Sgo1 accumulated at centromeres in cells expressing GFP-CENP-B-HDAC1, but not in those expressing GFP-CENP-B-HDAC1 H141A (Fig. 5b). Consistent with these observations, H2A-pT120 levels in the chromatin-bound fraction were also restored to that of wild-type cells by CENP-B-HDAC1 (Fig. 5c). Ultimately, centromere-tethered HDAC1 significantly suppressed PSCS in RSF1 KO cells, whereas CENP B-HDAC1-H141A failed to do so (Fig. 5d). Taken together, these data indicate that centromeric tethering of HDAC1 bypasses the functional requirement of RSF1 in centromeric cohesion protection. This genetic suppression experiment further supports the notion that RSF1 recruits HDAC1 to centromeres to deacetylate H2A K118, allowing Bub1 to phosphorylate H2A at T120. Our results provide the first evidence for a specialized function of HDAC1 in chromosome segregation during mitosis.

### The acetyltransferase Tip60 mediates H2A-K118 acetylation in mitosis

**Fig. 6:**
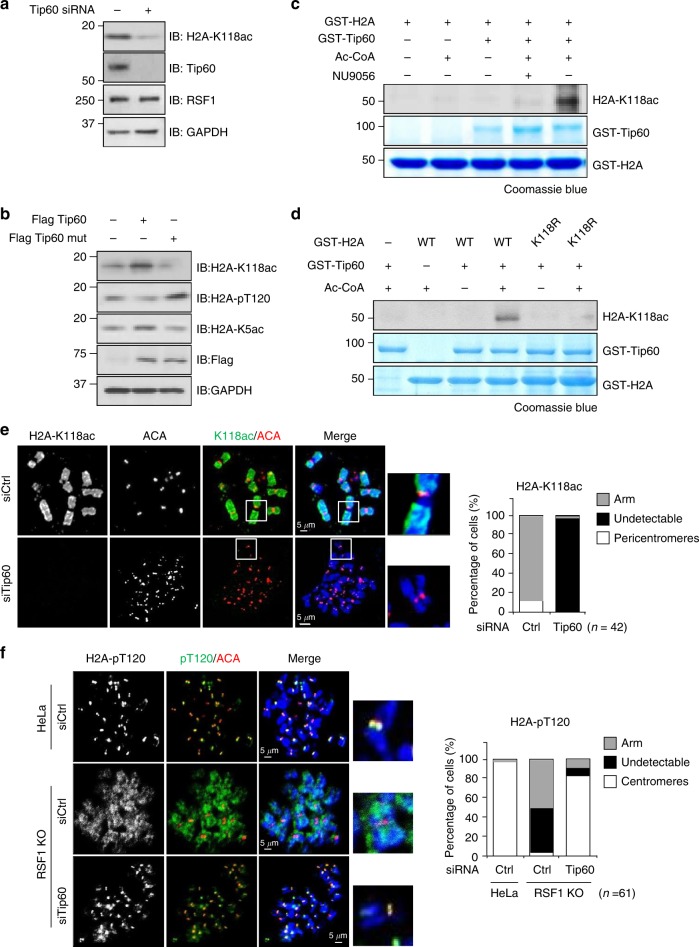
The acetyltransferase Tip60 mediates H2A-K118 acetylation. **a** HeLa cells were transfected with siTip60 and arrested in mitosis and mitotic cell lysates were subjected to immunoblotting. **b** Lysates of HeLa cells transfected with Flag-Tip60 WT or catalytic mutant of Tip60 were blotted with indicated antibodies. **c** Recombinant GST-H2A protein was incubated with purified GST-Tip60 in the presence or absence of Ac-CoA and NU9056, Tip60 specific inhibitor, for in vitro acetylation assay. The acetylation levels of H2A were analyzed with an anti-H2A-K118 acetylation antibody. **d** Recombinant GST-H2A WT or K118 mutants were incubated with purified GST-Tip60 in the presence of Ac-CoA for in vitro acetylation assay. The acetylation levels of H2A were analyzed with an anti-H2A-K118 acetylation antibody. **e** Mitotic HeLa cells depleted of Tip60 were stained with anti-ACA (red), anti-H2A-K118ac (green) antibodies. The percentages of cells exhibiting the arm or centromeric level of H2A-K118ac are shown. Scale bar, 5 μm. **f** RSF1 WT or knockout HeLa cells were transfected with control or Tip60 siRNA and stained with anti-ACA and anti-H2A-pT120 antibodies. The percentages of cells exhibiting the arm or centromeric level of H2A-pT120 are shown. Scale bar, 5 μm

We showed that H2A-K118ac staining was present throughout the mitotic chromosome arms in HeLa cells (Fig. 2g). Because Tip60 was recently shown to induce Aurora B acetylation during mitosis^41^, we tested whether Tip60 was responsible for the acetylation of H2A-K118. Depletion of Tip60 by siRNA greatly decreased H2A-K118ac levels (Fig. 6a). Similar result was obtained in cells treated with NU9056, a Tip60-specific inhibitor (Supplementary Fig. 5a). Tip60 acetylates H2A at K5^42^. We found that Tip60, but not its acetyltransferase-dead mutant, increased the acetylation of both H2A-K118 and H2A-K5 (Fig. 6b). An in vitro acetylation assay showed that purified Tip60 increased the level of H2A-K118ac, and this effect was blocked by the presence of NU9056 (Fig. 6c). In addition, the Tip60-mediated acetylation of H2A-K118 was not observed in the H2A-K118R mutant (Fig. 6d). Consistent with these observations, H2A-K118ac on chromosome arms was not observed in Tip60-depleted cells (Fig. 6e). Thus, we conclude that Tip60 acetylates H2A-K118 during early mitosis.

Because Tip60-mediated acetylation of H2A-K118 hinders Bub1-mediated phosphorylation of H2A-T120 and Sgo1 accumulation, we predicted that depletion of Tip60 would restore Sgo1 accumulation in RSF1 KO cells. KD of Tip60 by itself did not affect centromeric H2A-pT120 and Sgo1 accumulation in HeLa cells (Supplementary Fig. 5c, d). We figured that in the absence of Tip60, H2A K118 deacetylation is maintained and thus, H2A-T120 phosphorylation and Sgo1 localization at centromeres are not interrupted. Strikingly, KD of Tip60 in RSF1 KO cells almost completely restored H2A-pT120 (Fig. 6f) and Sgo1 (Supplementary Fig. 5f) deposition at centromeres because deacetylation activity by RSF1-mediated HDAC1 is of no use in these cells. Therefore, a major function of RSF1 is to recruit HDAC1 to centromeres, where it antagonizes Tip60-mediated acetylation of H2A- K118, which allows Sgo1 accumulation and centromeric cohesion protection.

## Discussion

**Fig. 7:**
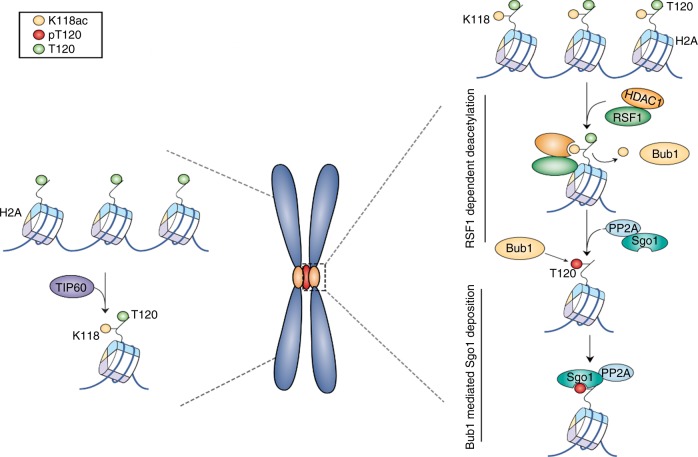
A proposed model for sister chromatid cohesion by RSF1. In our model, Tip60 contributes to maintaining H2A-K118ac at chromosome arms and RSF1-bound HDAC1 at centromeres counteracts it, allowing the phosphorylation of H2A at T120 by Bub1. Green circle represents H2A-T120 and red circle is its phosphorylated form (H2A-pT120). Yellow circle represents acetylation of H2A at Lys 118 (H2A-K118ac)

The role of histone modifications in genome regulation has been widely reported in recent years^35,43,44^. Relatively less information is available regarding the functions of histone modifications in mitotic chromosomes in human cells. Here, we have uncovered a critical function of the RSF1 chromatin remodeler in Sgo1 localization through coordinating the crosstalk of different histone modifications at the mitotic centromeres as shown in our proposed model (Fig. 7). Tip60 mediates H2A-K118 acetylation (yellow circle), which inhibits Bub1-dependent H2A-T120 phosphorylation. RSF1 recruits HDAC1 to mitotic centromeres. HDAC1-mediated deacetylation of H2A-K118 then enables H2A-T120 phosphorylation (red circle: H2A-pT120) by Bub1. Sgo1 binding to H2A-pT120 and to cohesin protect centromeric cohesion, which is required for faithful chromosome segregation. Thus, RSF1 regulates the acetylation/deacetylation balance of H2A on mitotic chromosomes in human cells.

RSF1 depletion causes the loss of Sgo1 and H2A-pT120 at centromeres (Fig. 1b, c). Bub1 generates the H2A-pT120 mark at centromeres during early mitosis and provides a docking site for Sgo1^26–29^. Centromeric loss of H2A-pT120 in RSF1 KO cells therefore results in the inability to recruit Sgo1. However, Bub1 localization and kinase activity are intact in these cells (Fig. 1d, e). In addition, the HDAC1-mediated deacetylation of H2A-K118 is required for the phosphorylation of H2A-T120. This suggests that an important mitotic function of RSF1 is to recruit HDAC1 to centromeres. The crucial physiological function of RSF1 in the HDAC1-mediated deacetylation of H2A-K118 at centromeres is further supported by its tight correlation with the occurrence of PSCS. Expression of RSF1 mutants retaining HDAC1 interaction (Fig. 4f and Supplementary Fig. 4d) or centromere tethering of HDAC1 (Fig. 5d) in RSF1 KO cells significantly suppresses PSCS, confirming the importance of this mechanism.

In more than 50% of RSF1 KO cells, Sgo1 is completely lost from chromosomes; however, approximately 40% of those cells showed the spread of Sgo1 and H2A-pT120 to chromosome arms (Fig. 1b, c). This is accompanied by an inverse relationship between H2A-pT120 and H2A-K118ac at both centromeres and chromosome arms (Fig. 2f, g). Tip60 contributes to maintaining H2A-K118ac at chromosome arms of human mitotic cells (Fig. 6e). RSF1-bound HDAC1 at centromeres counteracts it, allowing the phosphorylation of H2A at T120 by Bub1 (Figs. 4 and 5). Therefore, RSF1 maintains the histone marks at centromeres and also affects these marks at chromosome arms (Fig. 7). Interestingly, a recent report showed that malonylation on H2A in yeasts also prevented Bub1-mediated phosphorylation and Sgo1 accumulation^45^. Thus, histone modifications at the H2A-K118 would affect the Bub1-mediated phosphorylation in both humans and yeasts but they may use different histone modifications. In response to RSF1 depletion, HDAC1 could not be localized at centromere, instead, it was dispersed to chromosome arms (Fig. 3b) and promotes the deacetylation of H2A-K118ac (Figs. 2g and 3e). This in turn enables Bub1 phosphorylation of H2A-T120 and Sgo1 localization to chromosome arms, as observed in RSF1 KO cells (Fig. 1b, c). Loss of Tip60 by itself in mitotic cells does not significantly affect Sgo1 and H2A-pT120 deposition at centromeres (Supplementary Fig. 5), despite the fact that Tip60 is shown to accumulate at pericentric heterochromatin^46^ and affects the acetylation of Aurora B^41^. We speculate that H2A-K118ac by Tip60 at chromosome arms may inhibit Bub1 phosphorylation of H2A-T120 and limits the spread of Sgo1 to chromosome arms.

It is of note that RSF1 depletion not only abolishes the enrichment of HDAC1, H2A T120 phosphorylation, and Sgo1 at centromeres; at the same time, all three concentrate at chromosome arms to an extent that exceeds their amount at centromeres under normal conditions (Fig. 5c, Supplementary Figs. 1e and 3a). It was previously shown that conditions in which proper Bub1 kinetochore targeting is impaired result in the spread of the H2A-pT120 signal and/or Sgo1 displacement along chromosome arms^29^. It can be postulated that HDAC1 and Sgo1 may bind the entire chromatin with low binding affinity but centromeric accumulation of these proteins is mediated by more specific regulation with high binding activity. Thus, loss of proper signaling necessary for centromeric accumulation result in the re-displacement of these proteins to chromosome arms. Moreover, RSF1 may somehow suppress HDAC1 binding to chromosome arms in normal conditions and thus, loss of RSF1 leads to hyper-accumulation of HDAC1 at chromosome arms (Fig. 3b, Supplementary Fig. 3a). In these conditions, H2A-K118 acetylation is absent at chromosome arms (Fig. 2g), which increases the chance for phosphorylation of H2A-T120 (Fig. 5c), leading to the hyper-accumulation of H2A-T120 and Sgo1 in RSF1 KO cells.

In conclusion, we have identified RSF1 as a critical chromatin remodeling component involved in the coordination of centromeric processes important for chromosome segregation, as well as kinetochore-microtubule attachment^11^. RSF1 does so by toggling a molecular switch that installs the proper histone marks at centromeres and chromosome arms. It will be interesting to further investigate how RSF1 itself is recruited to centromeres and whether RSF1 contributes to the formation of a centromere-specific chromatin domain in collaboration with other centromere-associated proteins.

## Methods

### Cell culture and treatments

HeLa (ATCC, CCL2™) and HeLa Tet-On (Invitrogen, #R71407) were maintained in high-glucose DMEM supplemented with 10% FBS (Invitrogen). Mycoplasma contamination was tested by MycoFluor™ Mycoplasma Detection Kit (Invitrogen, #M7006) in all cell lines. RSF1 KO HeLa cells were generated by transcription activator-like effector nucleases (TALEN)-mediated genome engineering^47^ and the RSF1-specific TALEN plasmids composed of the florescence surrogate reporter (pRG2S) system were purchased from ToolGen, Inc. (Korea). At 48 h post-transfection of RSF1-specific TALEN plasmids with pRG2S, the cells expressing green fluorescence were sorted under FACS (FACS Vantage, BD Biosciences)^11^. To obtain cells synchronized at prometaphase, cells were treated with 100 ng/ml of paclitaxel (Sigma-Aldrich) or 100 nM of nocodazole (Sigma-Aldrich) for 12–16 h and collected by gentle shake-off.

### Transfection and RNA interference

The transfection of plasmids or siRNA oligonucleotides was carried out using polyethylenimine (PEI, Polysciences) or Lipofectamine 2000 (Invitrogen) following the manufacturer’s protocol^10^. The siRNA oligonucleotide sequences are as follows: human RSF1 #1: 5′-UCGAAACGAGUUGGCUGAGACUCUU-3′; RSF1 #2: 5′-GGAAAAUGUCAAACCCAUU-3′; and human SNF2H: 5′-AUAGCUCUUCAUCCUCCUCUU-3′; human HDAC1: 5′-CUAAUGAGCUUCCAUACAA-3′; human Tip60: 5′-GGACAGCUCUGAUGGAAUA-3′.

### Plasmids and purification of recombinant proteins

The plasmid encoding human H2A WT were cloned into the pGEX6p-1 vector to produce N-terminal GST fusion proteins. Site-directed mutagenesis was carried out on the GST-H2A plasmid using Muta-Direct™ Site Directed Mutagenesis Kit (iNtRON Biotechnology) to change Lys 118 and Lys 119 to Arg or Glu (K118R, K118Q, K119R, K119Q). These vectors were transformed into bacteria, and expression of GST-H2A proteins was purified from bacterial lysates using glutathione sepharose 4B (GE Healthcare)^29^. The mutagenesis primer sequences are as follows: H2A K118R-F: 5′-CGTGCTGCTGCCTAGGAAAACTGAGAGCC-3′; H2A K118R-R: 5′-GGCTCTCAGTTTTCCTAGGCAGCAGCACG-3′; H2A K118Q-F: 5′-CCGTGCTGCTGCCTCAGAAAACTGAGAGC-3′; H2A K118Q-R: 5′-GCTCTCAGTTTTCTGAGGCAGCAGACG-3′; H2A K119R-F: 5′-GCTGCTGCCTAAGAGAACTGAGAGCCACC-3′; H2A K119R-R: 5′-GGTGGCTCTCAGTTCTCTTAGGCAGCAGC-3′; H2A K119Q-F: 5′-GTGCTGCTGCCTAAGCAAACTGAGAGCCAC-3′; H2A K119Q-R: 5′-GTGGCTCTCAGTTGCTTAGGCAGCAGCAC-3′. Plasmids encoding V5-tagged full-length and deletion mutants of RSF1 (N2, N3, C1, and C2) were kindly provided by Dr. Ie-Ming Shin (Johns Hopkins Medical School). Flag-tagged, full length, and deletion constructs of HDAC1 were kindly provided by Dr. Jae-Hong Seol (Seoul National University). Site-directed mutagenesis was carried out on the RSF1-V5 C1 plasmid using Muta-Direct™ Site Directed Mutagenesis Kit (iNtRON Biotechnology) to change L1244, Ser1245, Ser1246, Ser1247, and E1248 to Ala. Recombinant baculoviruses of GST-RSF1 WT, C2 and N3, were created using Gateway® system (Invitrogen). Briefly, RSF1 cDNA was subcloned into the pDEST20 destination vector using LR Clonase^TM^ (Invitrogen). The pDEST20-RSF1 bacmid was transfected into Sf9 cells and the supernatant containing recombinant virus (GST-RSF1) was collected after 4 days. The original CenpB-Bub1-GFP plasmid were provided by Dr. Hongtao Yu (University of Texas Southwestern Medical Center, USA). CENPB-HDAC1-GFP was constructed by replacing with the HDAC1 cDNA fragment after digestion with Nhe I/Asc I.

### Immunoprecipitation and in vitro binding assays

For immunoprecipitation, cells were lysed using a sonicator (EpiShear™ Probe Sonicator, Active motif, 120 Watt, 20 KHz) in E1A buffer (50 mM Tris-HCl [pH 7.4], 150 mM NaCl, 0.1% NP-40, 1 mM DTT, 5 mM EDTA) containing protease and phosphatase inhibitors (1 mg/ml aprotinin, 1 mg/ml leupeptin, 5 mM NaF, 0.5 mM Na_3_VO_4_). Protein lysates were immunoprecipitated with indicated antibodies for 12 h at 4 °C under constant rotation. The protein–antibody complex was further incubated with protein A-Sepharose beads (GE Healthcare) for 1 h 30 min at 4 °C and the immune complex were washed and subjected to immunoblotting. For immunoblotting, the following antibodies were used: mouse anti-RSF1 (Upstate, 1:1000, #05-727), rabbit anti-RSF1 (abcam, 1:1000, ab109002), rabbit anti-POGZ (abcam, 1:1000, ab171934), rabbit anti-HDAC1 (GeneTex, 1:1000, GTX100513) and mouse anti-HDAC1 (Cell signaling, 1:1000, #5356s/Active motif, 1:1000, #39531), mouse anti-CENP-A (abcam, 1:1000, ab13939), rabbit anti-H2A K118ac (PTM biology, 1:2000, PTM-173), rabbit anti-H2A pT120 (Active motif, 1:2000, #39391) antibodies. For in vitro binding assays, recombinant proteins of GST-H2A, GST-RSF1, and GST-Tip60 were prepared as described in the above. The proteins were incubated for 2 – 12 h at 4 °C under constant rotation and beads-bound immune complexes were washed for four times and subjected to immunoblot analysis. Uncropped images of western blotting are shown in Supplementary Figure 6.

### In vitro kinase assay

Recombinant GST-fused H2A proteins (K118A, K118Q, K119A, K119Q) or were incubated with either human GST-Bub1 in the kinase buffer (20 mM HEPES, 0.14 M NaCl, 3 mM KCl, 5 mM MgCl_2_, pH 7.4) with 2 μCi of γ^32^P‐ATP (PerkinElmer^TM^) for 30 min at 30 °C. The reactions were terminated by adding 6× SDS sample buffer followed by heating 100 °C for 5 min. The proteins were separated on gradient SDS-polyacrylamide gel, and the incorporation of ^32^P was visualized by autoradiography. Uncropped images of western blotting are shown in Supplementary Figure 6.

### In vitro substrate kinase assay

Peptide kinase assays were incubated with recombinant GST-Bub1 in 50 mM Tris [pH 7.5], 0.1 mM EGTA, 10 mM MgCl_2_ and 0.1 mM γ^32^P‐ATP for 20 min at 30 °C in the presence of the peptide substrate.

### Cell fractionation

Mitotic cells were lysed with fractionation buffer I (10 mM Tris [pH 8.0], 25 mM KCl, 5 mM MgCl2, 0.5% NP40, 1 mM DTT, 1 mg/ml aprotinin, 1 mg/ml leupeptin, 5 mM NaF, 0.5 mM Na_3_VO_4_) and incubated on ice for 5 min. The supernatant (S1) and pellet (P1) were obtained after centrifugation at 1300×*g* for 5 min at 4 °C. The S1 fraction was refined by high-speed centrifugation at 20,000×*g* for 10 min at 4 °C and the supernatant was used as a soluble cytosolic fraction (S2). The insoluble pellet (P1) was washed twice and lysed with fractionation buffer II (10 mM Tris [pH 8.0], 500 mM NaCl, 0.1% NP-40, 5 mM EDTA, 1 mg/ml aprotinin, 1 mg/ml leupeptin, 5 mM NaF, 5.5 mM Na_3_VO_4_) by sonication (Active motif, 120 Watt, 20 KHz). After centrifugation at 1700×*g* for 5 min at 4 °C, the chromatin bound nuclear fraction (supernatant) was obtained. The concentrations of lysates were normalized by Bradford assay (Bio-Rad Laboratories), and lysates were analyzed by immunoblotting. Uncropped images of western blotting are shown in Supplementary Figure 6.

### Chromosome spreading

HeLa cells were treated with 100 ng/ml of nocodazole for 4 h and floating mitotic cells were collected by gentle shake-off. The cells were incubated in KCl (75 mM) buffer for 10 min at room temperature and centrifuged at 200×*g* for 5 min using Cytospin (Hanil Science, Korea). The cells were fixed with 4% paraformaldehyde for 15 min and then permeabilized with 0.5% Triton X-100 for 10 min. The fixed cells were blocked with 3% bovine serum albumin for 1 h at room temperature. The antibodies were used; mouse anti-RSF1 (Upstate, 1:100, #05-727), human anti-ACA (Immunovision, Inc, 1:2,000), rabbit anti-H2A K118ac (PTM biology, 1:100, PTM-173), rabbit anti-H2A pT120 (Active motif, 1:500, #39391), mouse anti-CENP-A (abcam, 1:100, ab13939), rabbit anti-POGZ (abcam, 1:100, ab171934), rabbit anti-KAT5/Tip60 (abcam, 1:100, ab23886). For image acquisition, Nikon A1R-A1 Confocal Microscope system with 60× 1.4 NA Plan-Apochromat objective (Nikon Instrument Inc.) or LSM710 with 63× 1.4 NA Plan-Apochromat objective (Carl Zeiss) were used and analyzed by the NIS elements C program or the ZEN 2011 program, and Image processing and quantification were carried out with ImageJ.

### In vitro acetylation assay

Recombinant GST-fused H2A was incubated with recombinant GST-Tip60 in 30 μl HAT buffer (20 mM Tris-HCl, pH 8.0; 10% glycerol; 100 mM NaCl; 1 mM DTT; 1 mM EDTA; 10 μM TSA; 10 mM NAM) containing 100 μM acetyl-CoA (Sigma) for 2 h at 37 °C. The reactions were terminated by adding 6× SDS sample buffer followed by heating at 100 °C for 5 min. The proteins were separated on gradient SDS-polyacrylamide gel and immunoblotted with indicated antibodies. Uncropped images of western blotting are shown in Supplementary Figure 6.

### Statistical analysis

In each result, the error bars represent the mean ± SEM from at least three independent experiments. The statistical significance was determined with two-sided unpaired Student’s *t*-test. *p* Values are indicated in the legends.

## Electronic supplementary material

Supplementary Information

Peer Review File

Description of Additional Supplementary Files

Supplementary Data 1

## Data Availability

The authors declare that all data supporting the findings of this study are available within the paper and its supplementary information files.
