# Supplementary material for: The chromatin remodeler RSF1 controls centromeric histone modifications to coordinate chromosome segregation
Source: Nat Commun. 2018 Sep 21;9:3848. doi: 10.1038/s41467-018-06377-w (PMC6155007; doi:10.1038/s41467-018-06377-w)

Supplementary information

The chromatin remodeler RSF1 controls centromeric histone modifications to coordinate chromosome segregation

Lee et. al

Supplementary Figure 1

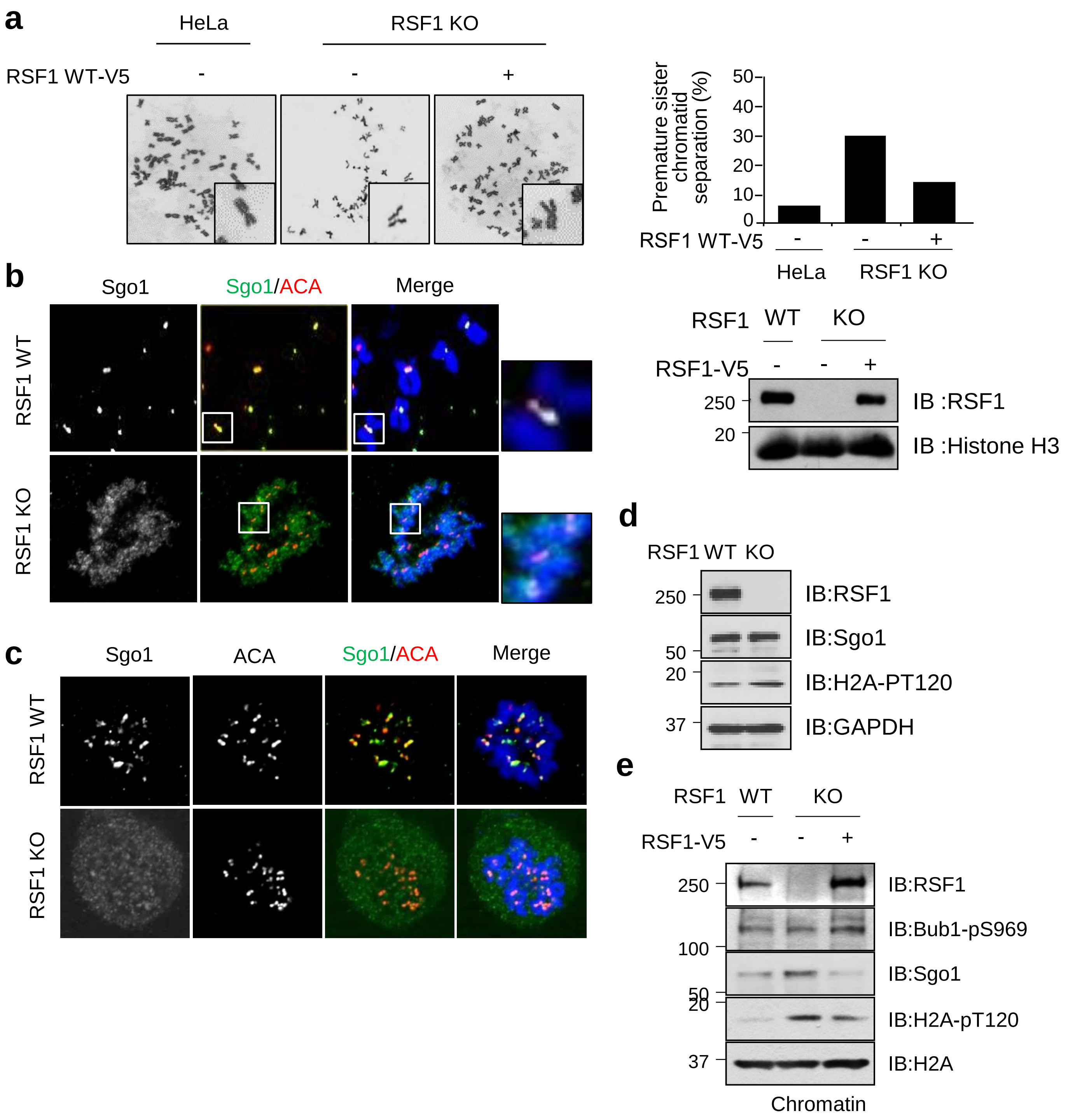

Supplementary Figure 1. RSF1 depletion hampers Sgo1 recruitment to kinetochores. (a) RSF1 knockout HeLa cells were transfected with pcDNA or RSF1-V5. Metaphase chromosome spreading was prepared and floating mitotic cells were stained with Giemsa. The Immunoblot at the bottom panel shows efficient reduction of RSF1 and SNF2H protein levels.**(b)** Immunofluorescence staining with anti-Sgo1 in RSF1 WT or RSF1 knockout HeLa cells. **(c)** Representative whole-cell images of Sgo1 (green) and ACA (red) staining in WT or RSF1 KO HeLa cells. **(d)** Western blot confirms the total level of Sgo1 in HeLa cells**.** **(e)** RSF1 WT plasmid were transfected into RSF1 KO cells and treated with Paclitaxel for 16h. The chromatin bound fractions were subjected to immunoblot analysis with indicated antibodies.

Supplementary Figure 2

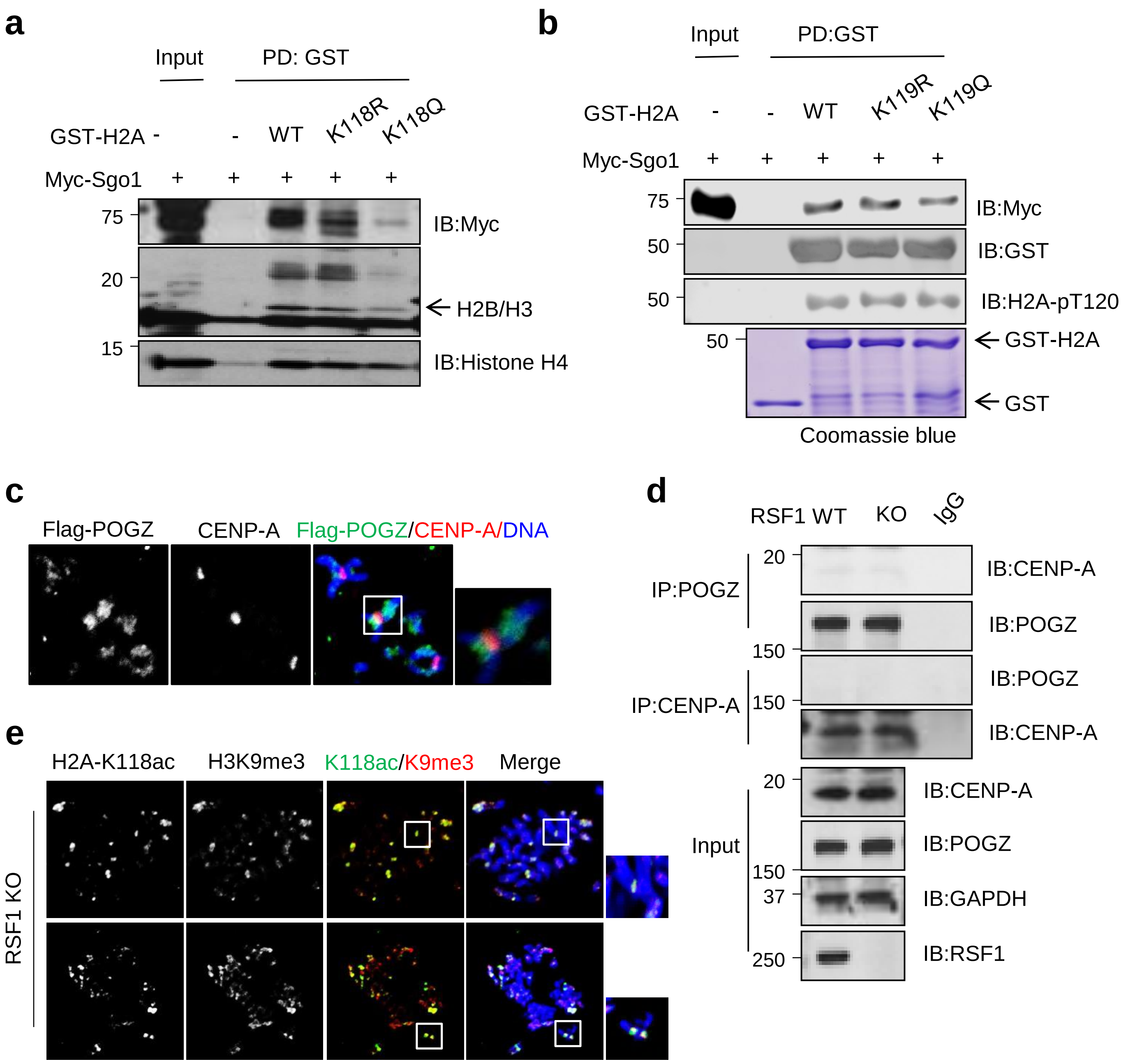

Supplementary Figure 2. H2A K119 does not affect interaction of between Sgo1 and H2A. (a) Recombinant GST-H2A WT or K118 mutants were incubated with Myc-tagged Sgo1 expressing mitotic whole cell lysates for 12h at 4 °C and subjected to immunoblot analysis with indicated antibodies. **(b)** Recombinant GST-H2A WT or K119 mutants were incubated with Myc-tagged Sgo1 expressing mitotic whole cell lysates for 12h at 4 °C and subjected to immunoblot analysis with indicated antibodies.**(c)** Chromosome spreads were prepared from Flag-POGZ transfected HeLa cells and stained with anti-Flag and anti-CENP-A antibodies. **(d)** RSF1 WT or knockout HeLa cell lysates were immunoprecipitated using anti-POGZ (chromosome arm protein) or anti-CENP-A (centromere protein) antibodies. Immunoprecipitaes were analysed by immunoblot using indicated antibodies. **(e)** H2A K118 acetylation and Histone H3K9me3 staining in chromosome spreads of mitotic RSF1 WT or KO HeLa cells.

Supplementary Figure 3

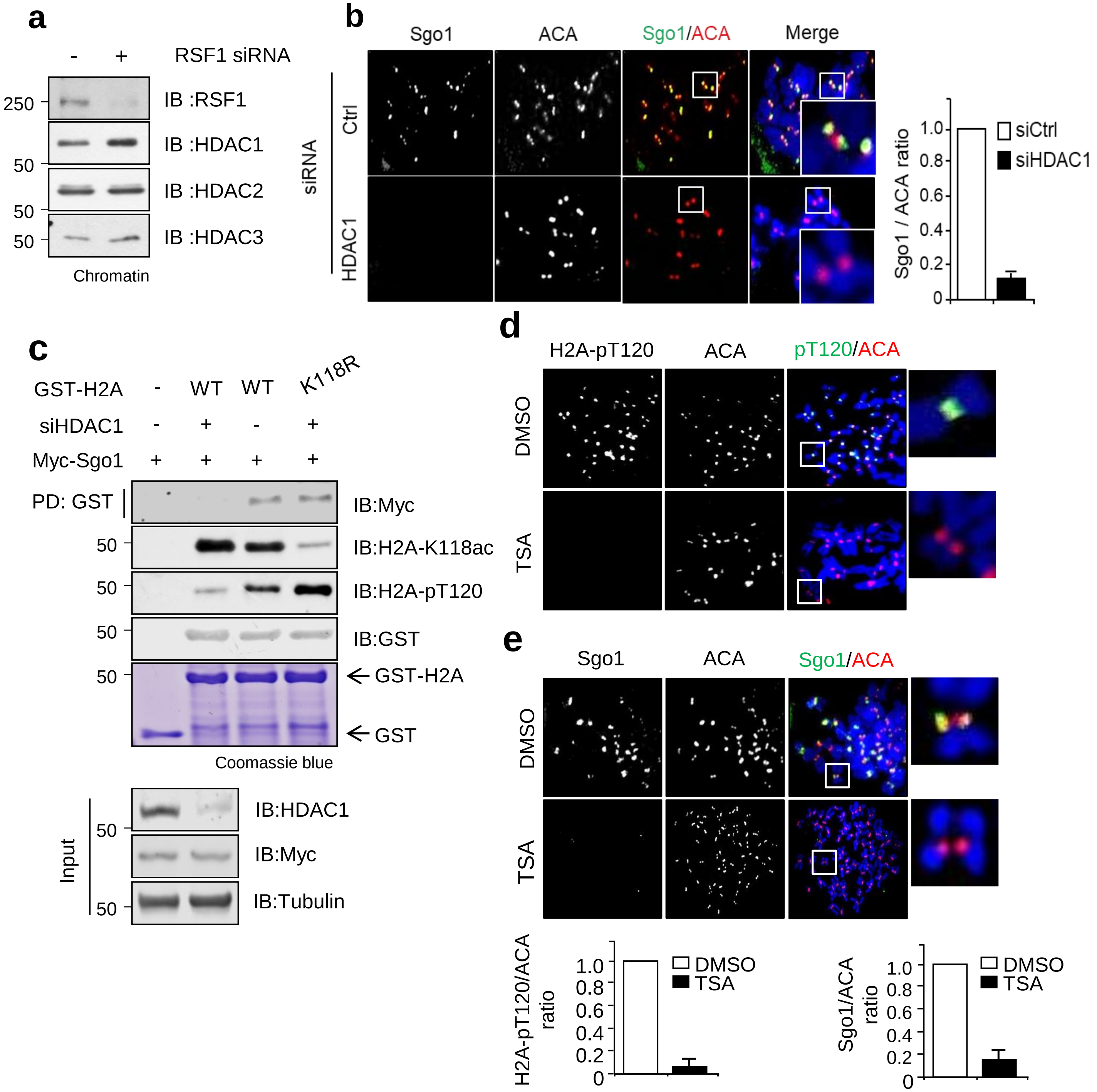

Supplementary Figure 3. HDAC1 regulates the acetylation of H2A-K118 in mitosis. (a) HeLa cells transfected with RSF1 siRNA were treated with paclitaxel for 16 h, and floating mitotic cells were obtained. Chromatin-bound fractions were subjected to immunoblotting with indicated antibodies. **(b)** HeLa cells transfected with HDAC1 siRNA were treated with nocodazole for 4 h, and floating mitotic cells were subjected to chromosome spread by staining with anti-Sgo1 antibody. **(c)** HeLa cells were co-transfected with Myc-Sgo1 and control siRNA or HDAC1 siRNA, and cells were treated with paclitaxel for 16h. Mitotic whole cell lysates were incubated with recombinant GST-H2A proteins for 12h at 4 °C and subjected to immunoblotting with indicated antibodies. **(d,e)** Nocodazole containing mitotic cells were treated with DMSO or 250nM TSA for 1 hr, Chromosome spread was performed using the anti-H2A-pT120 and anti-Sgo1 antibodies. The graph represents relative intensity of H2A-pT120 or Sgo1 against ACA at kinetochores.

Supplementary Figure 4

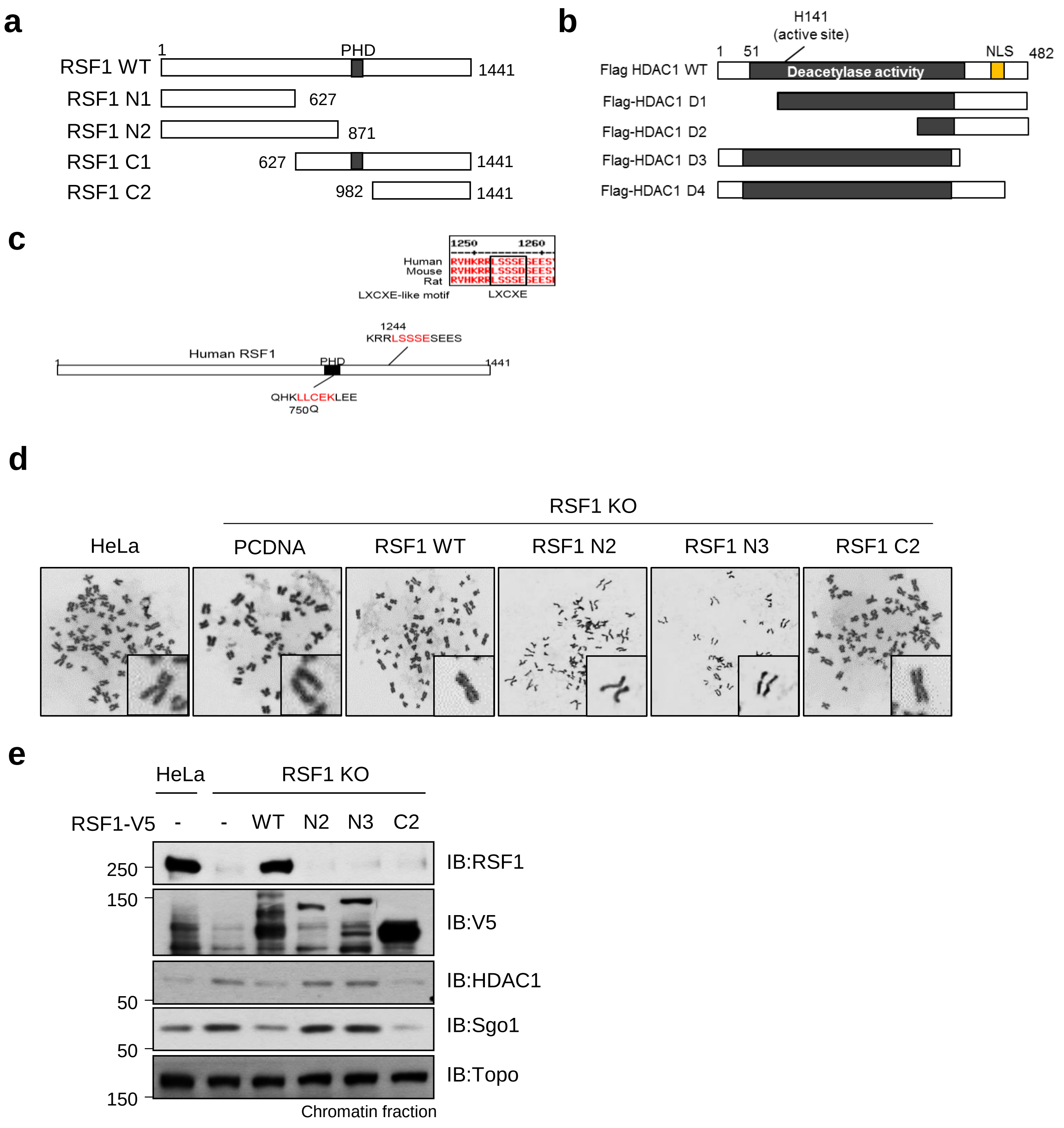

Supplementary Figure 4. RSF1 associates with HDAC1. (a) Schematic representation of the deletion mutants of RSF1 organization. **(b)** Schematic drawings of HDAC1 deletion mutants used in the manuscript. **(c)** Schematic drawing of domains and motifs of human RSF1, and the sequence alignment of vertebrate RSF1 with the conserved LXCXE-like motif. **(d)** RSF1 knockout cells were cotransfected with indicated RSF1-V5 constructs. Chromosome spreads of nocodazole treated cells stained with giemsa. **(e)** Chromatin fractions in RSF1 KO cells after reintroduction of indicated RSF1-V5 constructs and cell lysates were subjected to immunoblotting.

Supplementary Figure 5

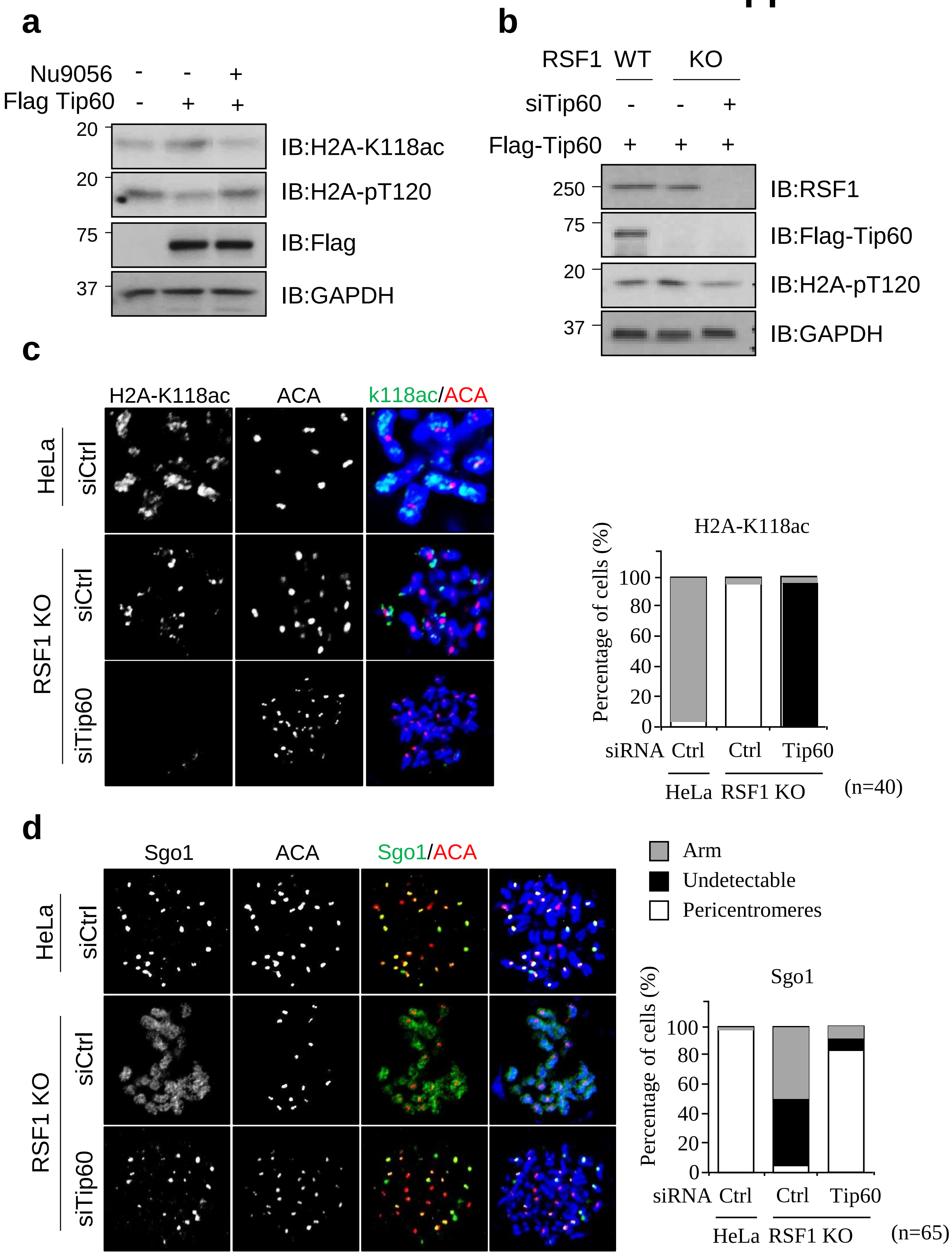

Supplementary Figure 5

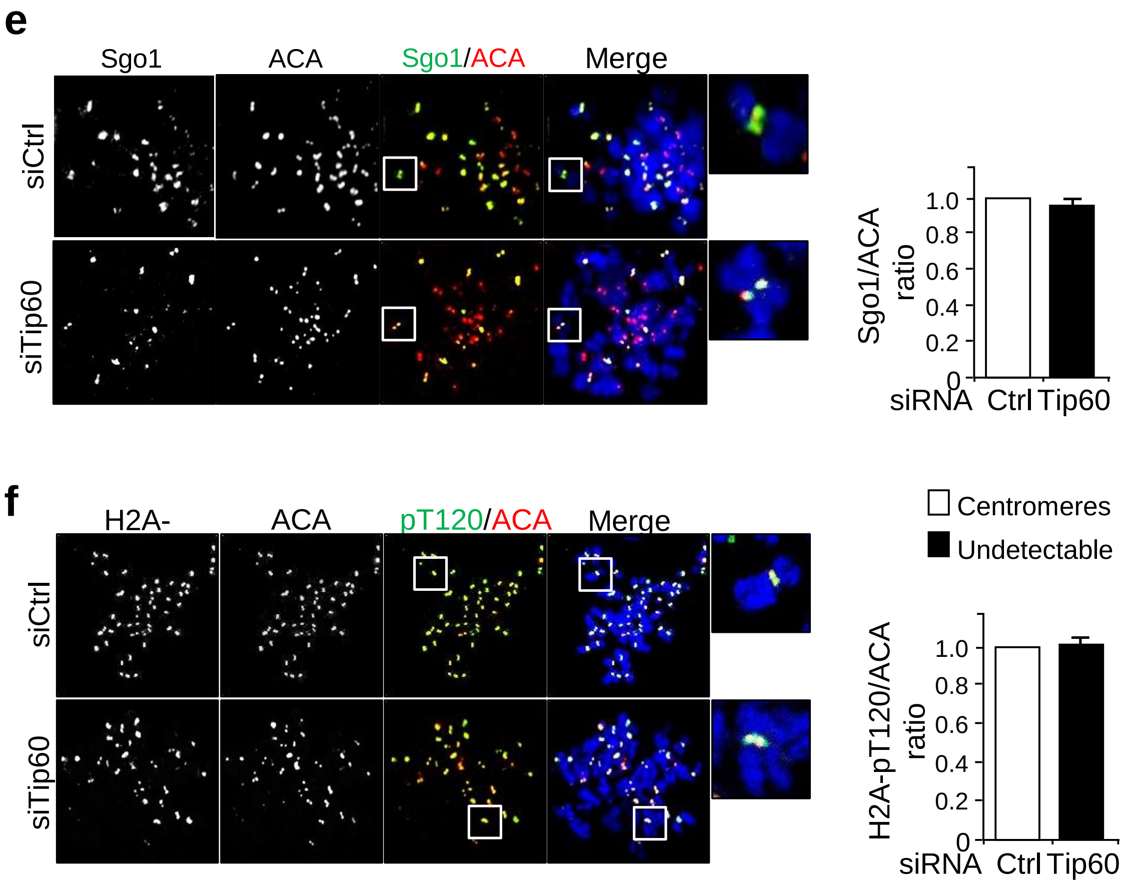

Supplementary Figure 5. Tip60 acetyltransferase acetylates the H2A-K118. (a) HeLa cells expressing Flag-Tip60 were synchronized by paclitaxel for 16 h, and the treated with DMSO or NU9056, Tip60 specific inhibitor, for 1 h. Mitotic whole cell lysates were subjected to immunoblotting with indicated antibodies. **(b)** Immunofluorescence staining of TIP60 (green) and ACA (red) in HeLa cells. Quantification of TIP60 fluorescence intensity (normalized to ACA) at kinetochores in HeLa cells. **(c)** Immunofluorescence staining of Sgo1 (green) and ACA (red) in Tip60 depleted HeLa cells. **(d)** HeLa cells transfected with Tip60 siRNA were subjected to chromosome spread and stained with anti-H2A-pT120 and anti-ACA antibodies. **(e,f)** RSF1 knockout HeLa cells were transfected with control or Tip60 siRNA and stained with anti-ACA (red) and anti-H2A-K118ac (green), anti-Sgo1 (green) antibodies. The percentages of cells exhibiting the arm or centromeric level of H2A-K118ac or Sgo1 are shown.

Supplementary Figure 6

Figure 1 a

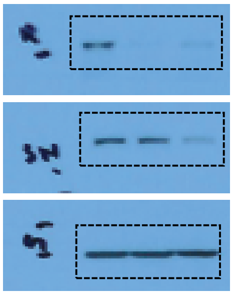

Figure 2 c

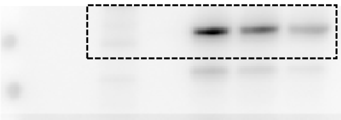

Figure 2 d

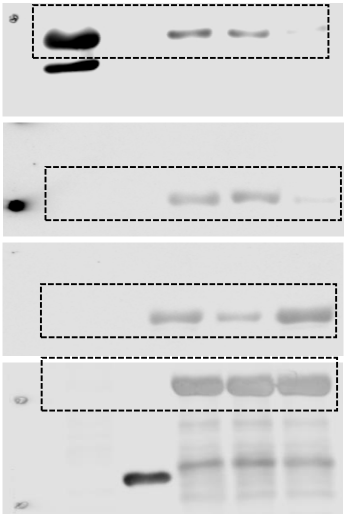

Figure 2 e

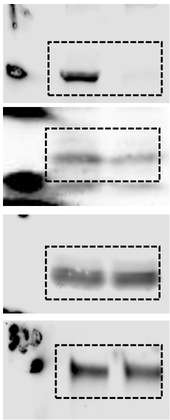

Figure 2 f

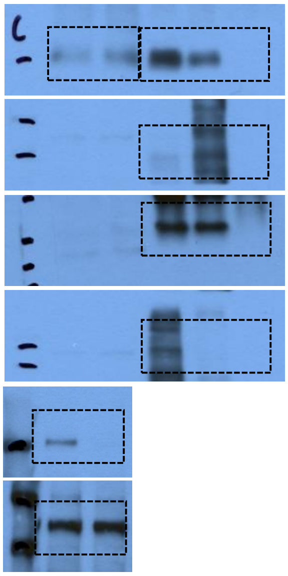

Figure 3 a

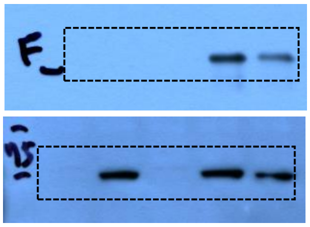

Figure 3 d

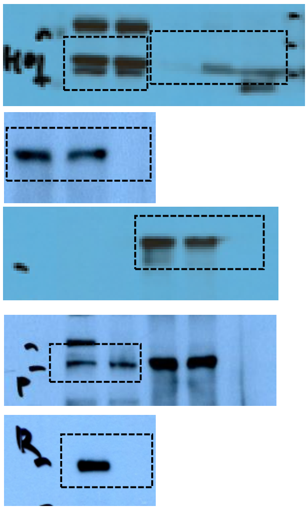

Figure 3 h

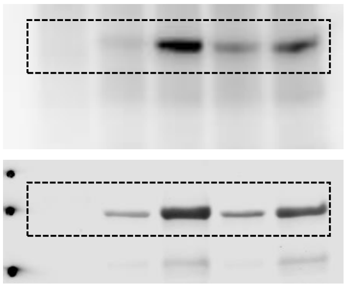

Figure 4 a

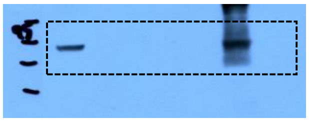

Figure 4 b

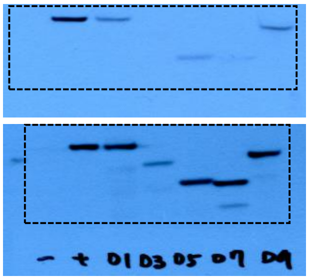

Supplementary Figure 6

Figure 4 c

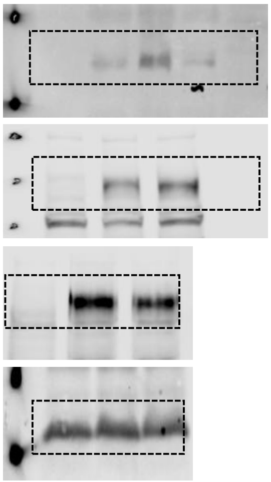

Figure 5 c

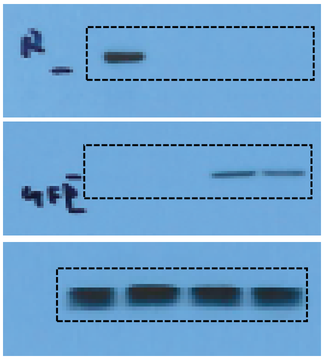

Figure 6 a

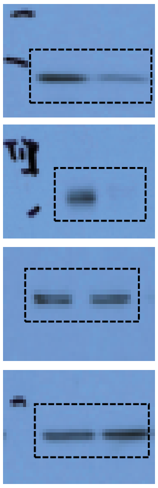

Figure 6 b

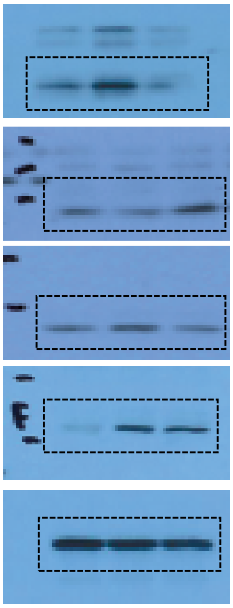

Figure 6 c

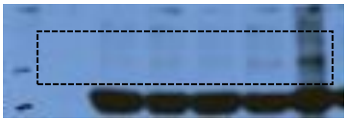

Figure 6d

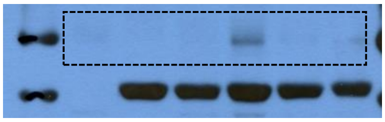

Figure S3 a

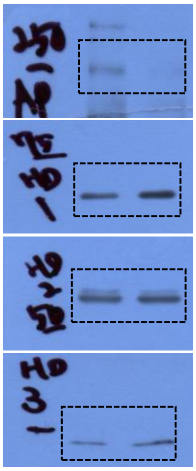

Figure S1 e

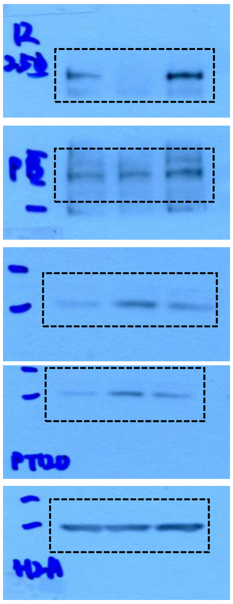

Supplementary Figure 6

Figure S3 c

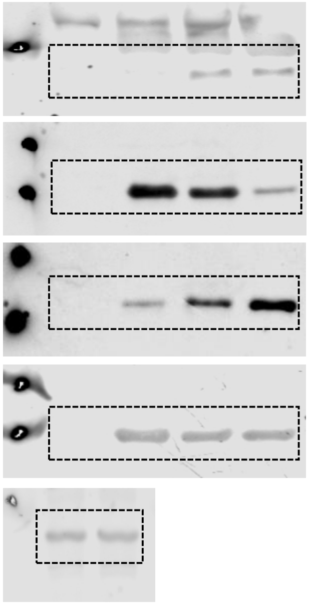

Figure S2 b

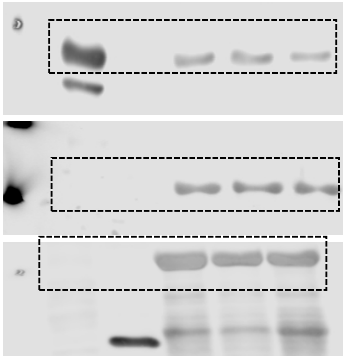

Figure S2 d

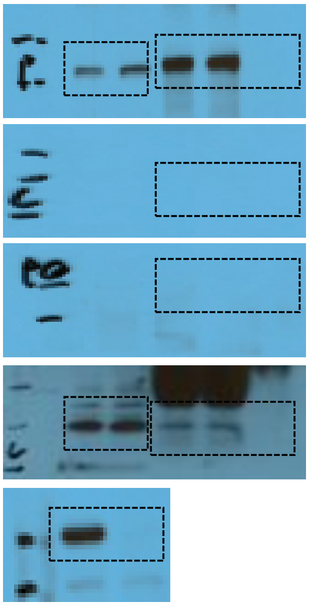

Supplement: Supplementary file 1 — Supplementary Information [file 41467_2018_6377_MOESM1_ESM.pdf]
