# Supplementary material for: The chromatin remodeler RSF1 controls centromeric histone modifications to coordinate chromosome segregation
Source: Nat Commun. 2018 Sep 21;9:3848. doi: 10.1038/s41467-018-06377-w (PMC6155007; doi:10.1038/s41467-018-06377-w)
Supplement: Supplementary file 3 — Description of Additional Supplementary Files [file 41467_2018_6377_MOESM3_ESM.pdf]

**Description of Additional Supplementary Files**

File Name: Supplementary Data 1
Description: The mass spectrometry data of RSF1-interacting proteins.
