# Supplementary material for: The chromatin remodeler RSF1 controls centromeric histone modifications to coordinate chromosome segregation
Source: Nat Commun. 2018 Sep 21;9:3848. doi: 10.1038/s41467-018-06377-w (PMC6155007; doi:10.1038/s41467-018-06377-w)
Supplement: Supplementary file 2 — Peer Review File [file 41467_2018_6377_MOESM2_ESM.pdf]

Reviewers' comments:

Reviewer #1 (Remarks to the Author):

Chromatin remodeling complex RSF1 localizes to centromeres and plays some roles for timely chromosome segregation. However, it was still unclear about essential function of RSF1 in centromeres. Authors in this paper found that Sgo1 localization was abolished, which resulted in premature sister chromatid separation in RSF1-depleated cells. As Sgo1 localization to centromeres is controlled by phosphorylation of T120 residue of histone H2A (H2A-T120), they examined this modification and found that acetylation of K118 of H2A inhibited H2A-T120, which caused mislocalization of Sgo1. They found that RSF1 interacts with HDAC1 at centromeres, which prevents acetylation of K118, leading to maintaining H2A-T120 and Sgo1 localization at centromeres.

I agree that this paper contains some new findings and is potentially interesting. However, I also found that some cytogenetic analyses are still immature and authors need substantial revision with additional experiments to address my specific concerns. My specific points are followings.

1. My major point is presentations showing premature sister chromatid separation in RSF-depleted cells. In Figure 1A, Figure 4F or Figure 5, they clearly showed premature sister chromatid separation. However, when they performed immunofluorescence analyses with various antibodies, sister chromatids are not always separated even in RSF1-depleted cells. For example, in Figure 1B, typical duplicated ACA signals on one chromosome (these two signals are very close, indicating no premature sister chromatid separation. I never see premature sister chromatid separation in other immunofluorescence data. Authors may do overstatement for analyses. This is the most essential point in this paper. Please clarify this point. I cannot believe data with current images
2. For immunofluorescence analyses, authors sometimes mentioned mislocalization to chromosome arm (Figure 1B, C, Figure 3, 4…). I did not understand how much populations of K118ac exists on chromosome arm and how mush populations of T120 phosphorylation exists on centromeres. Although I understand authors idea, more quantitative analyses are essential to draw their conclusion. Another method such as ChIP-seq may be necessary to say their conclusion
3. Concerning Figure 2G, authors concluded that H2AK118ac is enriched at centromeres on RSF1-KO cells. However signals are not detected in centromeres (Signals of ACA and K118ac are clearly distinct). I do not believe that H2AK118ac is enriched at centromeres on RSF1-KO cells.
4. As a minor point, merged images for pT120/GFP were shown in Figure 5A and B. Here, as pT120 localization is most important, they should show only pT120 signals in this column. It was sometimes difficult to see pT120 signals in these images.

Reviewer #2 (Remarks to the Author):

Review of the Nature Communications manuscript NCOMMS-18-07011-T by Ho-Soo Lee et al., entitled "The chromatin remodeler RSF1 controls centromeric histone modifications to coordinate chromosome segregation"

When metazoan cells undergo mitosis the sister chromatid cohesion mediating cohesin ring complex is removed from chromatin in two steps. While most cohesin from chromosome arms is displaced in a non-proteolytic manner during prophase, centromeric cohesin is protected from this so-called prophase pathway and removed only by separase-dependent cleavage at the metaphase-to-anaphase transition.

Cohesin's association with chromatin is dynamic even in interphase and subject to a tug-of-war between the anti-cohesion establishment activity of Wapl and its cohesion-promoting antagonist

sororin. Inactivation of the bulk of sororin by phosphorylation likely explains the massive dissociation of cohesin from chromatin in early mitosis. At centromeres sororin (and cohesin) is protected from phosphorylation (and ring opening) by Sgo1-PP2A. Sgo1 initially binds via its SGO-C box to Bub1-phosphorylated Thr120 of histone H2A at kinetochores. Following this local enrichment and its Cdk1-dependent phosphorylation at Thr346, Sgo1 is then handed over (by a transcription-requiring mechanism) to centromeric cohesin.

In the manuscript at hand the Cho and Yu labs report additional steps of the recruitment mechanism that ultimately leads to the localization of Sgo1 to kinetochores/centromeres. Starting from the observation that the chromatin remodeler RSF1 also concentrates at centromeres in mitosis, the authors report that acetylation of histone H2A at Lys118 by Tip60 prevents Bub1-dependent phosphorylation of histone H2A at Thr120. RSF1 interacts with HDAC1 and recruits it to centromeres where it de-acetylates H2A K118 to facilitate T120 phosphorylation and centromeric focussing of Sgo1-PP2A.

The authors establish an unexpected link between a chromatin remodeling factor and cellular measures to ensure proper sister chromatid segregation in mitosis. Their findings are novel and unexpected and the presented data appear to be of high quality.

Despite my overall fondness for this impressive body of work, I have the following major points of criticism:

1) If H2A K118 (de)acetylation operates upstream of H2A T120 phosphorylation, why then is T120 phosphorylation and Sgo1 localization normal in the absence of Tip60 (fig. S4) and in the absence of both RSF1 and Tip60 (fig. 6f)? In the absence of H2A K118 acetylation throughout the entire chromatin, according to the authors' model, Bub1 is expected to phosphorylate H2A also along chromosome arms. Does this mean that H2A K118 acetylation is not required to prevent H2A T120 phosphorylation on arms? The authors need to address this issue because otherwise several statements within their manuscript cannot be upheld.
In the discussion the authors write (line 292f): "On the other hand, H2A K118ac by Tip60 at chromosome arms inhibits Bub1 phosphorylation of H2A-T120 and limits the spread of Sgo1 to chromosome arms." What data support this statement?

2) RSF1 depletion not only abolishes the enrichment of HDAC1, H2A T120 phosphorylation and Sgo1 at kinetochores/centromeres; at the same time all three concentrate at chromosome arms to an extent that exceeds their amount at centromeres under normal conditions. This hyper-accumulation cannot simply be explained by the sole block of centromeric localization. Could it be that RSF1 somehow actively suppresses HDAC1 binding to chromosome arms? The fact that in the absence of RSF1 H2A K118 acetylation is only found at centromeres and missing from arms would be consistent with this assumption. The authors should discuss this issue in more detail then they do so far.

3) Unfortunately, the manuscript is poorly written and parts are sloppily assembled.
A (non-exhaustive) list of examples is following:

- line 59f: "During prophase in human cells, mitotic kinases phosphorylate cohesin..."
Cohesin is not at all introduced.

- line 99f: "...autophosphorylation at S969 were intact and not affected by RSF1 KO (Fig. 1e).... The autophosphorylation of Bub1 was not affected by the presence or absence of RSF1."
Redundancy.

- line 121f: "..., we generated an acetylation-dead H2A mutant (K118R) and an acetylation-mimetic H2A (K118Q) mutant, and expressed them in HeLa cells."
- line 130: "...cells overexpressing the acetylation-dead H2A-K119R mutant,..."

These statementes are wrong. According to the legend GST-tagged H2A variants were expressed in and purified from bacteria. The immobilized histone variants were then incubated with mitotic cell lysates, washed and finally analysed by immunoblotting for PTMs and associated proteins.

- line 126f: "Myc-Sgo1 in mitotic cell extracts failed to bind to the acetylationmimetic H2A-K118Q pull-down (PD) (Fig. 2d)." As this poor expression illustrates, the entire paragraph (lines 120-135) needs to be re-written to prevent misleading the reader and improve the language.

- line 146f: "Co-immunoprecipitation with anti-H2A-pT120 antibody precipitated CENP-A,...."
Poor expression.

- line 151f: "...co-immunoprecipitation with anti-H2A-K118ac antibody mainly co-precipitated POGZ,..."
Poor expression.

- line 186: "...indicated by the level of GST-H2A pulled down from HDAC1-depleted cells (Fig. 3h)."
Again, this description of the experiment is faulty.

- line 187f: "Phosphorylation of the acetylation-dead H2A-K118R mutant was not affected by the absence of HDAC1 (Fig. 3h and Supplementary Fig. 2c).
The authors cannot say this because they to not analyse H2A-K118R in the presence of HDAC1. They can merely say that phosphorylation of H2A-K118R in the absence of HDAC1 was nearly as effective as of H2A-WT in the presence of HDAC1.

- line 514: "p values are indicated in the legends."
They are not!

- line 589f: "Quantification of the percentage of premature sister chromatid separation in HeLa cells were quantified."

- figure 2a: What is the sequence of the "starting" peptide? Where does it come from - H2A? This information is nowhere to be found.

- figure 2g: KO or RNAi? Labeling of IF and graph are contradictory.

- figure 3b: Magnified inset is missing in the top row.

- figure 3g: "siCon" instead of "siCtrl" as in the rest of the manuscript.

- figure 4e: Where are the RSF1 signals in the HeLa control (top row, second image from left)?

- figure 6c: Lane 1 seems to be missing in all three panels.

- figure 6g: As drawn, the model implies that Tip60 puts T120 in place....

- Several quantifications lack error bars (figure 1b, 1c, 2g, 4e, 6e, 6f, S1a, S4c, S4d). Does this mean the corresponding experiments were conducted only once?

4) As a rationale for the identification of a HDAC1-binding deficient RSF1 variant, it is stated that "the interaction of HDAC1 with its binding partners occurs through a consensus binding motif [LXCXE]. We searched for the LXCXE motif in RSF1 and...". This makes no sense! It has been reported (but also questioned by others) that HDAC1 interacts via a LxCxE-like motif with the pocket domain of Rb. Thus, one would have to look for a pocket domain within RSF1 (which is

obviously not there) and not for a LxCxE-like motif because this is present in HDAC1. I therefore strongly suggest to remove the sentence: "These data demonstrate that RSF1 interacts with HDAC1 through an LXCXE motif,...".

In summary, while this manuscript has the potential to be a strong candidate for Nature Communications, it first needs major revisions. However, I would like to stress that only my # 1 will potentially involve additional experiments; all others points of criticism can be addressed in writing/by editing.

Reviewer #3 (Remarks to the Author):

The maintenance of centromeric cohesion is crucial for the faithful mitosis and genomic stability. The centromere and kinetochore localization of Sgo1 protein is essential for the centromeric cohesion meaintenace. Mechanistically, Sgo1 recruits PP2A phosphotase to count-act the kinase activity of Plk1 and Aurora B (towards cohesin complex). Biomedical researcher especially scientists on cell cycle and mitosis have strong interest in the regulation of sister chromotid cohesion.

In this study, Lee et al., identified chromatin remodeler RSF1, as a novel regulator to protect centromeric cohesion. Depletion of RSF1 caused mislocalization of Sgo1 and H2A-pT120. The authors demonstrate that acetylation of H2A-K118 by the acetyltransferase Tip60 suppresses H2A-T120 by Bub1. They further show that RSF1 recruites HDAC1 to centromere and HDAC1-meditaed deacetylation of H2A-K118 is a precondition for robust accumulation of H2A-pT120 and Sgo1 centromere localization. Overall, this study discovered RSF1 regulates the crosstalk of H2A phosphorylation and acetylation and contributes to the maintenance of precision of mitosis. This study provide novel knowledge of centromeric cohesion maintenance and encompasses an impressive amount of genetic and the cell biological work. I support the publication of this work after clarification of a few specific points.

Major points:
- The direct mass spectrum evidence of acetylation modification of H2A K118 should be provided.

- The detailed characterization of RSF1 KO cell line should be presented.

- The knock-down efficiency of RSF1 siRNA should also be presented.

- Why Sgo1 is undetectable in 50% cells of RSF1 KO or KD (Fig. 1b, 1c)? The Sgo1 protein level should be analyzed by Western blot for these cells. Maybe it is better to provide an intact cell immunofluorescene staining instead of chromosomal spread staining.

- A few Western blot images quality is low such as Fig. 5c, Fig. 6b. It is better to repeat to get the convincing data.

- Lines 95-107, the authors investigated the RSF1-H2A(pT120)-Sgo1 signaling axis in this report. Given the involvement of Cdk-dependent phosphorylation of human Sgo1 at T346 (pT346; Liu et al., Nat. Cell Biol. 2013), the authors may also want to check to see if Cdk-dependent phosphorylation of Sgo1 (pT346) is altered in the RSF1 RNAi cells to better understand the context-dependent RSF1-Sgo1 signaling pathway and its role for the protection of centromeric

cohesion during mitosis.

- Lines 120-131, and 468: the authors mentioned H2A (K118R) and (K119R) a lot of times in the main text of their MS, but these mutants were never described in the Method section. Instead, the H2A mutants (K118A, K119A, K119Q) in the method section were never mentioned in the main text. Thus, the authors looked carefully through their MS to correct this issue.

Minor issues
- line 166, "Although the transcriptional function of HDAC1 has been studied extensively, its subcellular localization and function in mitosis remain unknown." Actually, a few papers had reported the related information. These publications should be cited and discussed. I listed a few publications at below.

Dynamic phosphorylation of Histone Deacetylase 1 by Aurora kinases during mitosis regulates zebrafish embryos development. Loponte S, et al., and Chiocca S. Sci Rep. 2016 Jul 26;6:30213.

Dynamic distribution of HDAC1 and HDAC2 during mitosis: association with F-actin. He S, Khan DH, Winter S, Seiser C, Davie JR. J Cell Physiol. 2013 Jul;228(7):1525-35.

HDAC1 inactivation induces mitotic defect and caspase-independent autophagic cell death in liver cancer. Xie HJ, Noh JH, Kim JK, Jung KH, Eun JW, Bae HJ, Kim MG, Chang YG, Lee JY, Park H, Nam SW. PLoS One. 2012;7(4):e34265.

MBD3 and HDAC1, two components of the NuRD complex, are localized at Aurora-A-positive centrosomes in M phase. Sakai H, Urano T, Ookata K, Kim MH, Hirai Y, Saito M, Nojima Y, Ishikawa F. J Biol Chem. 2002 Dec 13;277(50):48714-23.

- Lines 217-233, the authors mentioned the GFP-CENP-B-HDAC1 construct, but they did not elaborate how they made the construct in the method section.

Response to reviewer comments (comments from reviewers in **blue,** responses in **black**)**:**

Chromatin remodeling complex RSF1 localizes to centromeres and plays some roles for timely chromosome segregation. However, it was still unclear about essential function of RSF1 in centromeres. Authors in this paper found that Sgo1 localization was abolished, which resulted in premature sister chromatid separation in RSF1-depleated cells. As Sgo1 localization to centromeres is controlled by phosphorylation of T120 residue of histone H2A (H2A-T120), they examined this modification and found that acetylation of K118 of H2A inhibited H2A-T120, which caused mislocalization of Sgo1. They found that RSF1 interacts with HDAC1 at centromeres, which prevents acetylation of K118, leading to maintaining H2A-T120 and Sgo1 localization at centromeres.

I agree that this paper contains some new findings and is potentially interesting. However, I also found that some cytogenetic analyses are still immature and authors need substantial revision with additional experiments to address my specific concerns. My specific points are followings.

1. My major point is presentations showing premature sister chromatid separation in RSF-depleted cells. In Figure 1A, Figure 4F or Figure 5, they clearly showed premature sister chromatid separation. However, when they performed immunofluorescence analyses with various antibodies, sister chromatids are not always separated even in RSF1-depleted cells. For example, in Figure 1B, typical duplicated ACA signals on one chromosome (these two signals are very close, indicating no premature sister chromatid separation. I never see premature sister chromatid separation in other immunofluorescence data. Authors may do overstatement for analyses. This is the most essential point in this paper. Please clarify this point. I cannot believe data with current images.

→→ We thank the reviewer for the keen and constructive suggestions. We agreed that we made the reviewer confused for that point. In the revised manuscript, we replaced the representative images of immunofluorescence staining of RSF1 KO cells with premature sister chromatid separation throughout the whole figures (Fig. 1b-1e, Fig.4d&e, Fig. 5a&b). For optimized immunofluorescence staining on mitotic chromosomes, we have been using a 'cytospin' centrifugation for chromosome spreads. Unlike HeLa cells, chromosomes of RSF1 KO HeLa cells got easily entangled after cytospin, probably due to high frequency of premature chromatid separation. In these cases, it made us difficult to carry out immunofluorescence staining properly and we might choose the images with intact ACA dots because all the analyses were carried out based on ACA dots. We appreciated the reviewer's keen points and replaced the representative images. We also repeated the experiments to verify your suggested points and reached the same conclusion.

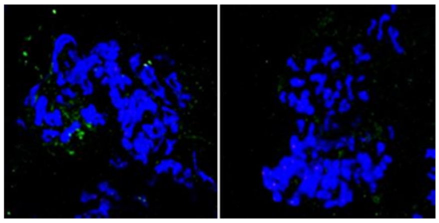

entangled chromosomes in RSF1 KO cells

Fig.1 b

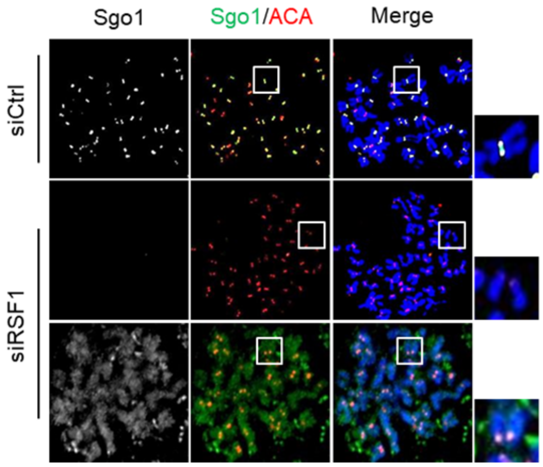

Fig.1 c

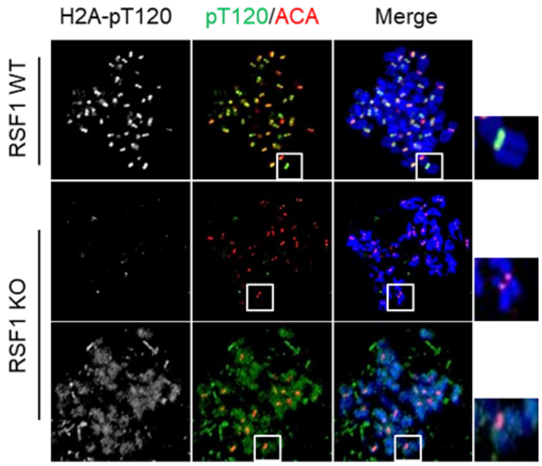

Fig.1 d

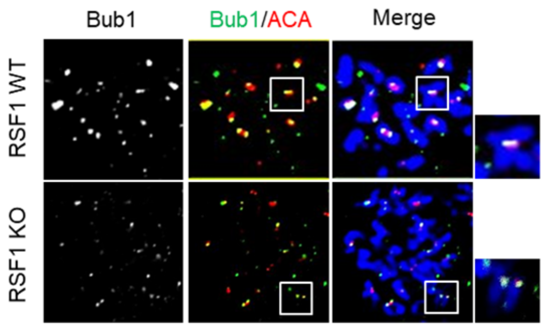

Fig.1 e

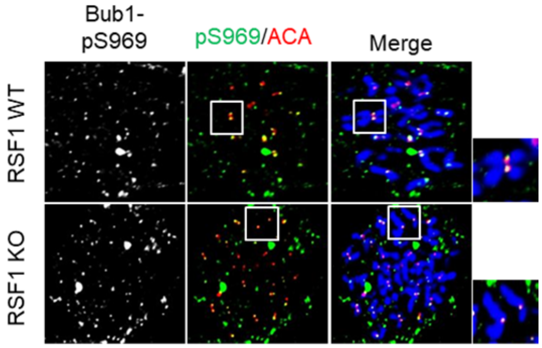

2. For immunofluorescence analyses, authors sometimes mentioned mislocalization to chromosome arm (Figure 1B, C, Figure 3, 4…). I did not understand how much populations of K118ac exists on chromosome arm and how mush populations of T120 phosphorylation exists on centromeres. Although I understand authors idea, more quantitative analyses are essential to draw their conclusion. Another method such as ChIP-seq may be necessary to say their conclusion

→ All the immunofluorescence staining data were shown with quantification of cell numbers in the original manuscript. We had tried the ChIP-seq the same as the reviewer's suggestions but we concluded that Chip-seq at human mitotic centromeres was technically unreliable (See below) and was impossible with currently available H2A-K118ac antibody. Instead, we developed co-immunoprecipitation experiments with anti-POGZ (chromosome arm-specific protein; *Nat Cell Biol* 12:719, 2010) antibody or centromere specific anti-CENP-A antibody (Supplementary Fig. 2d, Fig. 2f). As far as we know, this is the first biochemical approach to analyze proteins on centromeres from chromosome arms.

You may recognize that human centromeres contain an α-satellite array, which itself consists of ~500,000 copies of ~170-bp tandem repeats per haploid genome. Previously, a couple of papers showed the Chip-seq data at centromeres in interphase cells (*J Cell Biochem* 93:286, 2004; *J Biol Chem* 279:37175, 2004; *Mol Cell* 42:285, 2011). According to these information, we had carried out

Chip-seq experiments using the same Chr1 and Chr4 primers for centromeric repeats, SAT2 for pericentromeric repeats and GAPDH for chromosome arms. In addition to non-specific amplification with Chr1 primers (See below), currently available anti-H2A K118ac antibody did not provide reliable data. I would like to point out that we are able to generate other histone modification data under DNA damage conditions using Chip-seq analysis.

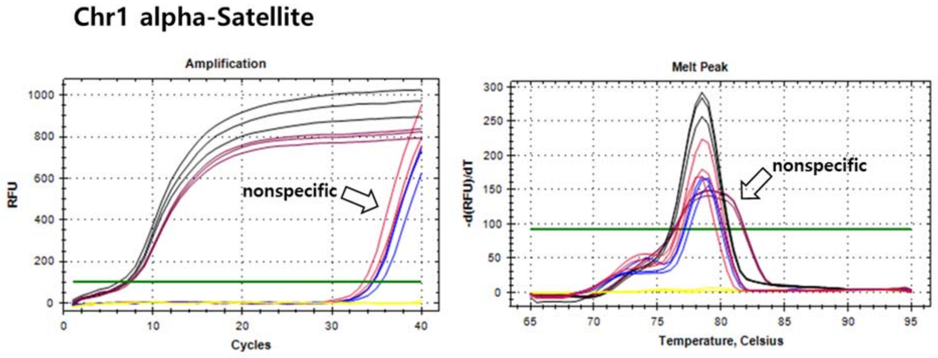

3. Concerning Figure 2G, authors concluded that H2AK118ac is enriched at centromeres on RSF1-KO cells. However, signals are not detected in centromeres (Signals of ACA and K118ac are clearly distinct). I do not believe that H2AK118ac is enriched at centromeres on RSF1-KO cells.

→ We also thank the reviewer for keen and constructive suggestions. As pointed by the reviewer, signals of ACA and K118ac were distinct in RSF1 KO cells. Because Tip60 acetyltransferase is reported to be concentrated on the pericentromeres (Mol Biol Cell, 27:599, 2016), we utilized H3K9me3 as a pericentromere marker and found that H2AK118ac was co-stained with H3K9me3 in RSF1 KO cells. We added the double-immunostaining data into the Supplementary Fig 2e and stated that 'Double-immunostaining with H3K9me3 as a pericentromeric marker revealed that H2AK118ac is enriched at pericentromeres of RSF1-KO cells' in the revised manuscript (lines 160-162).

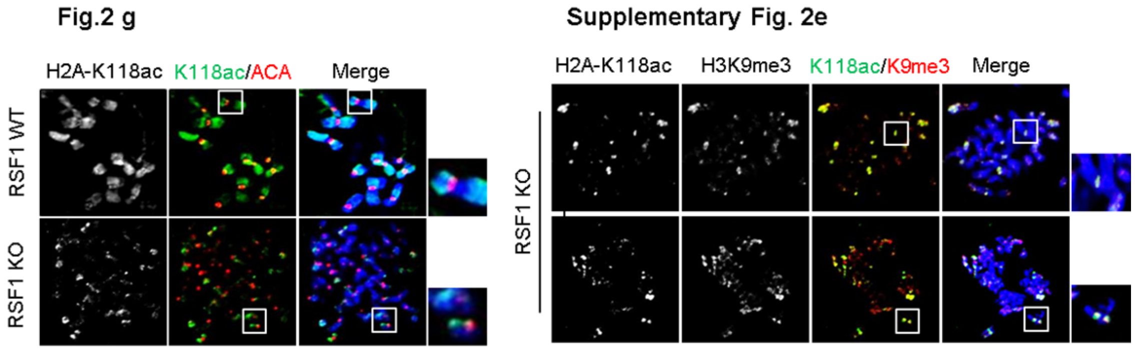

4. As a minor point, merged images for pT120/GFP were shown in Figure 5A and B. Here, as pT120 localization is most important, they should show only pT120 signals in this column. It was sometimes difficult to see pT120 signals in these images.

→ We showed the pT120-only signals in the first column of Figure 5a and b in the revised manuscript.

Fig.5 a,b

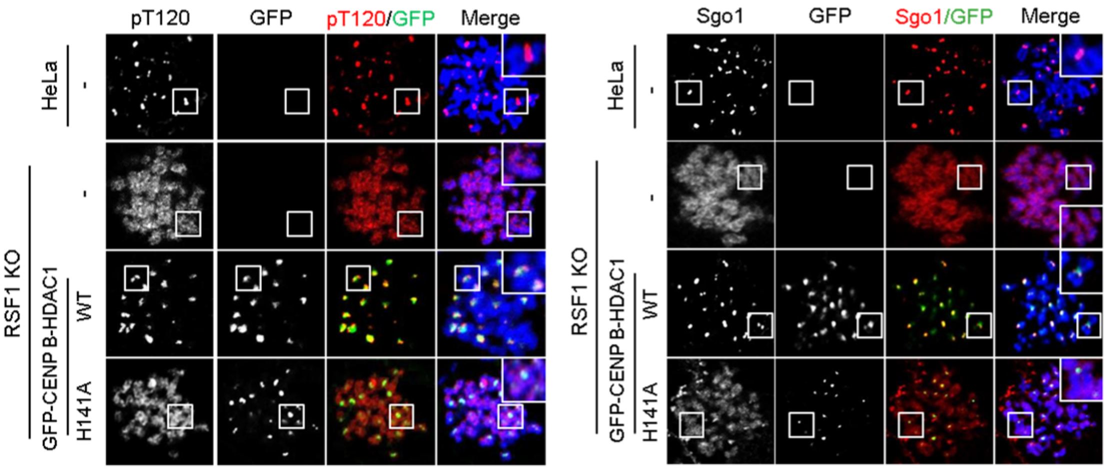

<u>Reviewer #2 (Remarks to the Author):</u>

Review of the Nature Communications manuscript NCOMMS-18-07011-T by Ho-Soo Lee et al., entitled "The chromatin remodeler RSF1 controls centromeric histone modifications to coordinate chromosome segregation"

When metazoan cells undergo mitosis the sister chromatid cohesion mediating cohesin ring complex is removed from chromatin in two steps. While most cohesin from chromosome arms is displaced in a non-proteolytic manner during prophase, centromeric cohesin is protected from this so-called prophase pathway and removed only by separase-dependent cleavage at the metaphase-to-anaphase transition.

Cohesin's association with chromatin is dynamic even in interphase and subject to a tug-of-war between the anti-cohesion establishment activity of Wapl and its cohesion-promoting antagonist sororin. Inactivation of the bulk of sororin by phosphorylation likely explains the massive dissociation of cohesin from chromatin in early mitosis. At centromeres sororin (and cohesin) is protected from phosphorylation (and ring opening) by Sgo1-PP2A. Sgo1 initially binds via its SGO-C box to Bub1-phosphorylated Thr120 of histone H2A at kinetochores. Following this local enrichment and its Cdk1-dependent phosphorylation at Thr346, Sgo1 is then handed over (by a transcription-requiring mechanism) to centromeric cohesin.

In the manuscript at hand the Cho and Yu labs report additional steps of the recruitment mechanism that ultimately leads to the localization of Sgo1 to kinetochores/centromeres. Starting from the observation that the chromatin remodeler RSF1 also concentrates at centromeres in mitosis, the authors report that acetylation of histone H2A at Lys118 by Tip60 prevents Bub1-dependent phosphorylation of histone H2A at Thr120. RSF1 interacts with HDAC1 and recruits it to centromeres where it de-acetylates H2A K118 to facilitate T120 phosphorylation and centromeric focussing of

Sgo1-PP2A.

The authors establish an unexpected link between a chromatin remodeling factor and cellular measures to ensure proper sister chromatid segregation in mitosis. Their findings are novel and unexpected and the presented data appear to be of high quality.

Despite my overall fondness for this impressive body of work, I have the following major points of criticism:

1) If H2A K118 (de)acetylation operates upstream of H2A T120 phosphorylation, why then is T120 phosphorylation and Sgo1 localization normal in the absence of Tip60 (fig. S4) and in the absence of both RSF1 and Tip60 (fig. 6f)? In the absence of H2A K118 acetylation throughout the entire chromatin, according to the authors' model, Bub1 is expected to phosphorylate H2A also along chromosome arms. Does this mean that H2A K118 acetylation is not required to prevent H2A T120 phosphorylation on arms? The authors need to address this issue because otherwise several statements within their manuscript cannot be upheld.

In the discussion the authors write (line 292f): "On the other hand, H2A K118ac by Tip60 at chromosome arms inhibits Bub1 phosphorylation of H2A-T120 and limits the spread of Sgo1 to chromosome arms." What data support this statement?

→ We thank the reviewer for the interesting and challenging comments. In the absence of Tip60, H2A K118 deacetylation is maintained and thus, H2A-T120 phosphorylation and Sgo1 localization at centromeres are not interrupted. Likewise, in the conditions of double-knockout of Tip60 and RSF1, H2A K118 deacetylation is maintained and thus, deacetylation activity by RSF1-mediated HDAC1 is of no use, showing normal H2A-T120 phosphorylation and Sgo1 localization at centromeres. The reviewer's input was added in the revised manuscript (lines 252-256).

→ Regarding to 2nd and 3rd comments, they are related to each other. In our model (Fig. 6g), Tip60 promotes H2A K118 acetylation throughout the entire chromatin. At centromeres, RSF1-interacting HDAC1 removes the acetyl group from the H2A K118ac, which allows the Bub1 mediated H2A-T120 phosphorylation and Sgo1 localization. In the absence of Tip60, H2A K118 acetylation throughout the entire chromatin was lost (Fig. 6e and Supplementary Fig. 4c). In these cells, the H2A pT120 and Sgo1 were entirely concentrated on centromeres (Supplementary Fig. 4e and 4f), not on chromosome arms. Because H2A K118 acetylation is lost on arms, effect of H2A K118 acetylation on H2A T120 phosphorylation there cannot be drawn. In the revised manuscript, we clarified our model of Fig. 6g by indicating histone modifications.

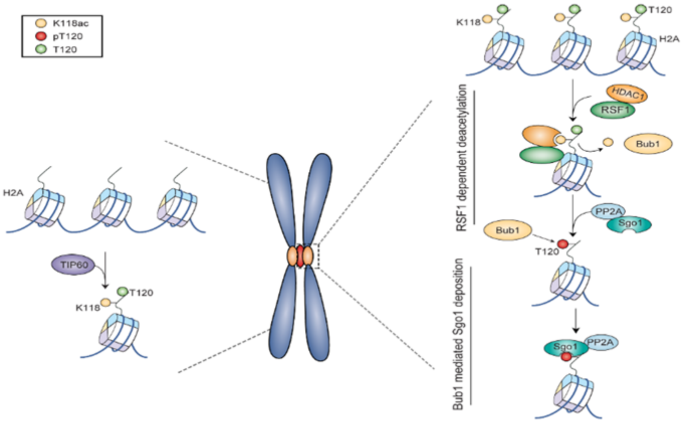

→ If the reviewer intended to ask ‘In the presence of H2A K118 acetylation……’ rather than ‘In the absence of H2A K118 acetylation…..’, we think that H2A K118 acetylation on arms may also provide additional barrier preventing H2A T120 phosphorylation because an inverse relationship between high H2A K118 acetylation and little H2A T120 phosphorylation on chromosome arms was observed (Fig. 3f and g). To further address it, we depleted the entire HDAC1 activity by TSA, an HDAC1 inhibitor and HDAC1 siRNA in RSF1 KO cells. Restoration of H2A K118 acetylation in RSF1 KO cells significantly alleviated the chromatin-bound fractions of H2A pT120 and Sgo1. All these results suggest that H2A K118 acetylation may prevent H2A T120 phosphorylation on the entire chromosomes. However, we could not completely exclude the possibility that cytosolic Bub1 may not be sufficient enough to efficiently phosphorylate H2A T120 on chromosome arms regardless of the status of H2A K118 acetylation. Because these are still our speculations, we wish to provide additional information to the reviewer. In the revised manuscript, we changed the sentence to ‘high H2A K118ac by Tip60 at chromosome arms may inhibit Bub1 phosphorylation of H2A-T120’(lines 295-296).

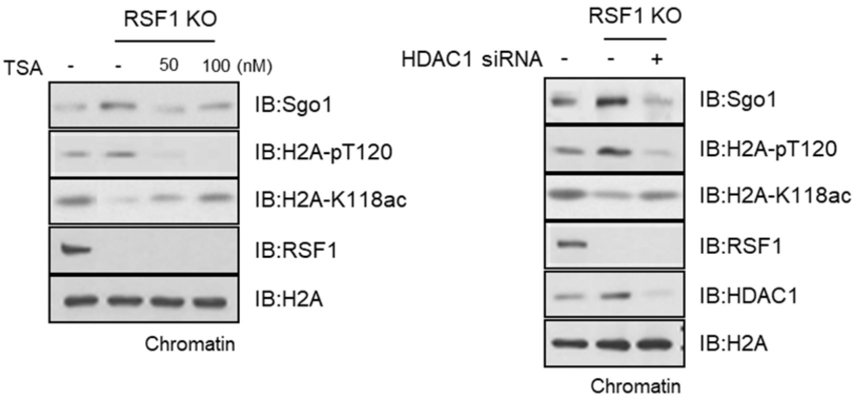

2) RSF1 depletion not only abolishes the enrichment of HDAC1, H2A T120 phosphorylation and Sgo1 at kinetochores/centromeres; at the same time all three concentrate at chromosome arms to an extent that exceeds their amount at centromeres under normal conditions. This hyper-accumulation cannot simply be explained by the sole block of centromeric localization. Could it be that RSF1 somehow actively suppresses HDAC1 binding to chromosome arms? The fact that in the absence of RSF1 H2A K118 acetylation is only found at centromeres and missing from arms would be consistent with this assumption. The authors should discuss this issue in more detail then they do so far.

→ Similar to our observation on hyper-accumulation of HDAC1, H2A pT120 and Sgo1 on

chromosome arms in RSF1 depleted cells, it was previously reported that conditions in which proper Bub1 kinetochore targeting is impaired result in the spread of the H2A-pT120 signal and/or Sgo1 displacement along chromosome arms (Current Biology, 15:353, 2005; 23:1917, 2013). We think that HDAC1 and Sgo1 may bind the entire chromatin with low binding affinity but centromeric accumulation of these proteins is mediated by more specific regulation with high binding activity. Thus, loss of proper signaling necessary for centromeric accumulation result in the re-displacement of these proteins along chromosome arms, leading to hyper-accumulation at the chromatin level. Because C-terminal of RSF1 strongly binds HDAC1 (Fig, 4), we cannot imagine how these RSF1-HDAC1 interactions work as the way by which RSF1 suppresses HDAC1 binding to chromosome arms. We added this issue into the Discussion of the revised manuscript (lines 297-306).

3) Unfortunately, the manuscript is poorly written and parts are sloppily assembled.

A (non-exhaustive) list of examples is following:

- line 59f: "During prophase in human cells, mitotic kinases phosphorylate cohesin..."

Cohesin is not at all introduced.

→ In the revised manuscript, we added the sentence introducing the cohesion ring complex and the whole paragraph is modified.

'Human sister chromatids at metaphase are primarily linked by cohesion ring complex at centromeres showing iconic X shape[12, 13]. Centromeres are specialized chromatin composed of highly repetitive α-satellite DNA in humans[14] and functional centromeres are marked by the presence of the centromere-specific histone H3-variant, CENP-A[15, 16]. ….. During prophase of human cells, cohesin from chromosome arms is displaced in a non-proteolytic manner. Mitotic kinases phosphorylate cohesin and its positive regulator sororin, and these phosphorylation events opens the cohesion ring complex and trigger the release of cohesin from chromosome arms[22, 23]. At centromeres, sororin and cohesion are protected from phosphorylation by the shugoshin1 (Sgo1) and protein phosphatase 2A (PP2A) complex[24,25].

- line 99f: "...autophosphorylation at S969 were intact and not affected by RSF1 KO (Fig. 1e)....

The autophosphorylation of Bub1 was not affected by the presence or absence of RSF1." Redundancy.

→ We changed it to 'By contrast, centromeric localization of Bub1 (Fig. 1d) and the autophosphorylation of Bub1 were not affected by the absence of RSF1'

- line 121f: "..., we generated an acetylation-dead H2A mutant (K118R) and an acetylation-mimetic H2A (K118Q) mutant, and expressed them in HeLa cells."

- line 130: "...cells overexpressing the acetylation-dead H2A-K119R mutant,..."

These statements are wrong. According to the legend GST-tagged H2A variants were expressed in and purified from bacteria. The immobilized histone variants were then incubated with mitotic cell lysates, washed and finally analysed by immunoblotting for PTMs and associated proteins.

- line 126f: "Myc-Sgo1 in mitotic cell extracts failed to bind to the acetylation mimetic H2A-K118Q pull-down (PD) (Fig. 2d)." As this poor expression illustrates, the entire paragraph (lines 120-135) needs to be re-written to prevent misleading the reader and improve the language.

→ As suggested by the reviewer, the entire paragraph was rewritten in the revised manuscript (lines 121-138).

'In H2A, two upstream lysine residues of K118 and K119 are well conserved among species (Fig. 2b). To test whether acetylation of K118 affected H2A-T120 phosphorylation by Bub1, we replaced the Lys 118 residue of H2A to Arg (H2A-K118R) in which acetylation site was disrupted. In addition, an acetylation mimicking mutant of H2A-K118Q (Lys118 to Gln) was generated. The GST-tagged H2A variants were purified in bacteria and subjected to *in vitro* kinase assay. As shown in Fig. 2c, phosphorylation on H2A-K118Q by Bub1 kinase was significantly reduced, whereas phosphorylation of the acetylation-dead mutant of H2A-K118R was similar to that of wild type H2A, suggesting that acetylation of the Lys118 inhibits the phosphorylation of H2A by Bub1. Next, the immobilized histone variants were incubated with mitotic cell lysates expressing Myc-Sgo1 and analyzed by immunoblotting for analysis of histone modifications and interactions. As expected, H2A-K118Q displayed a strong H2A-K118 acetylation with a weak H2A-T120 phosphorylation. And Sgo1 binding to the H2A-K118Q was also substantially reduced (Fig. 2d). We verified that immobilized H2A histone variants were tightly associated with other histones such as H2B, H3, and H4 (Supplementary Fig. 2a), indicating that GST-H2A was incorporated into nucleosomes in these experiments, which allows Sgo1 binding[30]. By contrast, H2A-pT120 phosphorylation of H2A-K119Q remained unchanged and Sgo1 binding to H2A-K119R and H2A-K119Q were as efficient as to wild type H2A (Supplementary Fig. 2b). Together, these findings indicated that H2A-pT120 phosphorylation and Sgo1 binding were modulated by the acetylation of neighboring H2A-K118, but not of H2A-K119.'

- line 146f: "Co-immunoprecipitation with anti-H2A-pT120 antibody precipitated CENP-A,...." Poor expression.

→ We changed it to 'To determine the distribution of H2A-pT120 on the chromatin), we immunoprecipitated H2A-pT120 in mitotic cell lysates and found that a subset of CENP-A, but not of POGZ, exists in a complex with H2A-pT120' in the revised manuscript.

- line 151f: "...co-immunoprecipitation with anti-H2A-K118ac antibody mainly co-precipitated POGZ,..." Poor expression.

→ We changed it to 'a major portion of H2A-K118ac was found to form a complex with POGZ in mitotic cells'

..

- line 186: "...indicated by the level of GST-H2A pulled down from HDAC1-depleted cells (Fig. 3h)."

Again, this description of the experiment is faulty.

→ We changed it to 'Consistent with these observations, in *in vitro* kinase assay Bub1-mediated phosphorylation of H2A-T120 was suppressed in the absence of HDAC1' in the revised manuscript.

- line 187f: "Phosphorylation of the acetylation-dead H2A-K118R mutant was not affected by the absence of HDAC1 (Fig. 3h and Supplementary Fig. 2c).

- The authors cannot say this because they to not analyse H2A-K118R in the presence of HDAC1.

They can merely say that phosphorylation of H2A-K118R in the absence of HDAC1 was nearly as effective as of H2A-WT in the presence of HDAC1.

→ We changed it to 'phosphorylation of H2A-K118R in the absence of HDAC1 was nearly as effective as of H2A-WT in the presence of HDAC1'

- line 514: "p values are indicated in the legends." They are not!

→ We added the *p*-value to the legend of Fig. 1a.

- line 589f: "Quantification of the percentage of premature sister chromatid separation in HeLa cells were quantified."

→We switched to 'Quantification of the percentage of premature sister chromatid separation in HeLa cells were shown'

- figure 2a: What is the sequence of the "starting" peptide? Where does it come from - H2A? This information is nowhere to be found.

→ We added following information into the Result (lines 114-116).

'The H2A substrate peptide [LLPKK(T)ESHH] of Bub1 is used as a starting peptide. Each amino acid positioned from -5 to +4 was switched from P (Pro) to Kme3 indicated at the Y-axis.'

- figure 2g: KO or RNAi? Labeling of IF and graph are contradictory.

→ We changed RNAi to RSF1 KO.

- figure 3b: Magnified inset is missing in the top row.

→ We added the magnified inset in the top row.

- figure 3g: "siCon" instead of "siCtrl" as in the rest of the manuscript.

→ We replaced 'siCon' to 'siCon' throughout the manuscript.

- figure 4e: Where are the RSF1 signals in the HeLa control (top row, second image from left)?

→ The representative image for RSF1 was replaced.

- figure 6c: Lane 1 seems to be missing in all three panels.

→ Lane 1 is removed from the figure.

- figure 6g: As drawn, the model implies that Tip60 puts T120 in place....

→ We put the Tip60 in the above of K118ac.

- Several quantifications lack error bars (figure 1b, 1c, 2g, 4e, 6e, 6f, S1a, S4c, S4d). Does this mean the corresponding experiments were conducted only once?

→ All the experiments were carried out at least 3 independent times, and we put the number of cells (>20 - 60) used for analysis.

4) As a rationale for the identification of a HDAC1-binding deficient RSF1 variant, it is stated that "the interaction of HDAC1 with its binding partners occurs through a consensus binding motif [LXCXE]. We searched for the LXCXE motif in RSF1 and...". This makes no sense! It has been reported (but also questioned by others) that HDAC1 interacts via a LxCxE-like motif with the pocket domain of Rb. Thus, one would have to look for a pocket domain within RSF1 (which is obviously not there) and not for a LxCxE-like motif because this is present in HDAC1. I therefore strongly suggest to remove the sentence: "These data demonstrate that RSF1 interacts with HDAC1 through an LXCXE motif,...".

→ We thank the reviewer for critical comments and corrected them in the revision (lines 201-207)

'We searched for any putative binding motifs in the C-terminal region of RSF1 and found that LSSSE as a LXCXE-like motif is well conserved among higher vertebrates (Supplementary Fig. 3c). It is shown that the LXCXE motif within the pocket domain of RB is crucial for interacting with various cellular and viral proteins[37]. These data demonstrate that the RSF1-HDAC1 is crucial for the prevention of premature chromosome segregation through the modulation of H2A-pT120.'

In summary, while this manuscript has the potential to be a strong candidate for Nature Communications, it first needs major revisions. However, I would like to stress that only my # 1 will potentially involve additional experiments; all others points of criticism can be addressed in writing/by editing.

→ We sincerely thank the reviewer for all the precise comments and constructive suggestions. We believe that all your comments significantly improved the manuscript.

Reviewer #3 (Remarks to the Author):

The maintenance of centromeric cohesion is crucial for the faithful mitosis and genomic stability. The centromere and kinetochore localization of Sgo1 protein is essential for the centromeric cohesion meaintenace. Mechanistically, Sgo1 recruits PP2A phosphotase to count-act the kinase activity of Plk1 and Aurora B (towards cohesin complex). Biomedical researcher especially scientists on cell cycle and mitosis have strong interest in the regulation of sister chromotid cohesion.

In this study, Lee et al., identified chromatin remodeler RSF1, as a novel regulator to protect centromeric cohesion. Depletion of RSF1 caused mislocalization of Sgo1 and H2A-pT120. The authors demonstrate that acetylation of H2A-K118 by the acetyltransferase Tip60 suppresses H2A-

T120 by Bub1. They further show that RSF1 recruites HDAC1 to centromere and HDAC1-meditaed deacetylation of H2A-K118 is a precondition for robust accumulation of H2A-pT120 and Sgo1 centromere localization. Overall, this study discovered RSF1 regulates the crosstalk of H2A phosphorylation and acetylation and contributes to the maintenance of precision of mitosis. This study provide novel knowledge of centromeric cohesion maintenance and encompasses an impressive amount of genetic and the cell biological work. I support the publication of this work after clarification of a few specific points.

Major points:

- The direct mass spectrum evidence of acetylation modification of H2A K118 should be provided.

→ We thank the reviewer for the suggestion and found that mass spectrum evidence of acetylation modification of H2A K118 has been already shown in different papers as well as in websites (*J Proteome Res* 5: 248, 2006; *Mol Cell Proteomics* 14: 2429, 2015; *PhosphoSitePlus*). However, biological function of the H2A K118 acetylation has never been addressed at all.

- The detailed characterization of RSF1 KO cell line should be presented.

→ RSF1 KO cell lines were previously established in our lab and described in detail (Nature Communications. 2015;6:7904). In the revised manuscript, we added this reference into the Result and Methods sections (lines 99, 436).

For the reviewer, we provided the information on RSF1 KO cell lines. We had applied the TALEN KO system to both HeLa (human cervical adenocarcinoma cells) and RPE1 (human retinal pigment epithelial cells). We were able to create viable RSF1 KO HeLa cells and however, RPE1 cells hardly grew on 96 well plates after sorting under FACS and died. We experienced that cancer cell lines generally tolerated stresses much better than immortalized cell lines such as RPE1. In fact, $Rsf1^{tm1b}$ KO mice exhibited complete preweaning lethality (European Mouse Mutant Archive).

- The knock-down efficiency of RSF1 siRNA should also be presented.

→ In Fig.1a of the revised manuscript, we added the immunoblotting data showing the knock-down efficiency of RSF1 siRNA. The knock-down efficiency of RSF1 siRNA is higher than 80%.

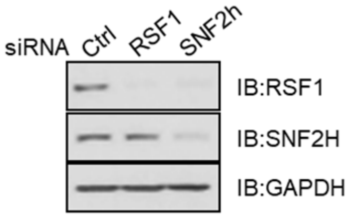

- Why Sgo1 is undetectable in 50% cells of RSF1 KO or KD (Fig. 1b, 1c)? The Sgo1 protein level should be analyzed by Western blot for these cells. Maybe it is better to provide an intact cell immunofluorescene staining instead of chromosomal spread staining.

→ As pointed out by the reviewer, total Sgo1 levels are not changed in RSF1 KO or KD cells as shown in Fig. 2e of the original manuscript. In immunofluorescence staining of RSF1 KO cells as shown below, diffused pattern of Sgo1 in RSF1 KO cells is detected in the cytosol. However, we experienced that some of the centromeric proteins including RSF1 are only detected in chromosome spread staining. The data was added into the Supplementary Figure 1 c, d of the revised manuscript (lines 94-96).

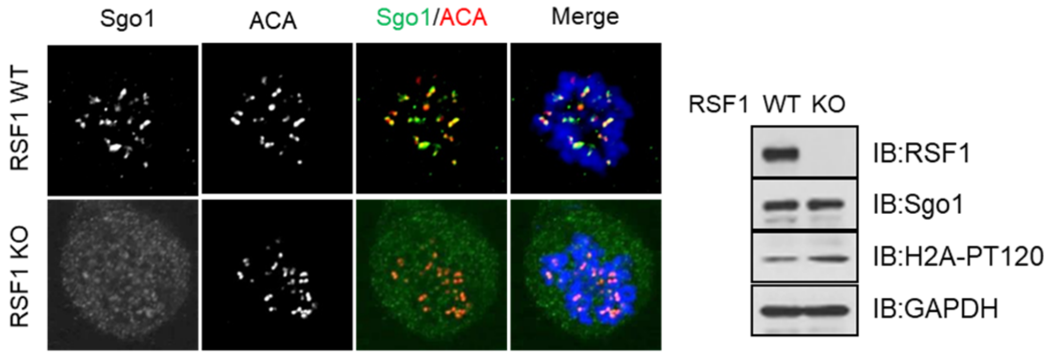

- A few Western blot images quality is low such as Fig. 5c, Fig. 6b. It is better to repeat to get the convincing data.

→ We replaced the data of as Fig. 5c and Fig. 6 in the revised manuscript.

Fig.5 c

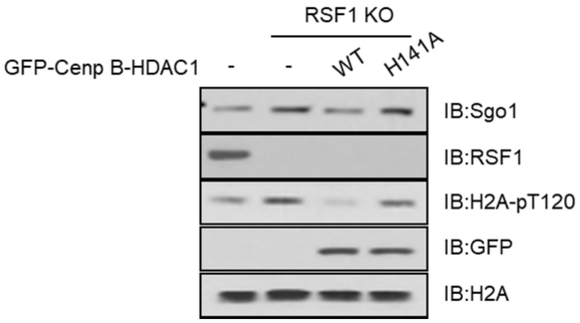

Fig.6 b

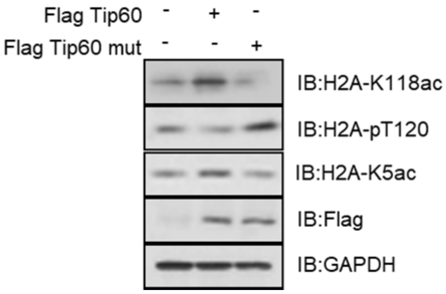

- Lines 95-107, the authors investigated the RSF1-H2A(pT120)-Sgo1 signaling axis in this report. Given the involvement of Cdk-dependent phosphorylation of human Sgo1 at T346 (pT346; Liu et al., Nat. Cell Biol. 2013), the authors may also want to check to see if Cdk-dependent phosphorylation of Sgo1 (pT346) is altered in the RSF1 RNAi cells to better understand the context-dependent RSF1-Sgo1 signaling pathway and its role for the protection of centromeric cohesion during mitosis.

→ In RSF1 KO cells, we found that phosphorylation of Sgo1 at T346 was reduced in immunoblotting and in chromosome spread immunostaining, suggesting that RSF1palys an important role in Sgo1 accumulation at centromeres/kinetochores.

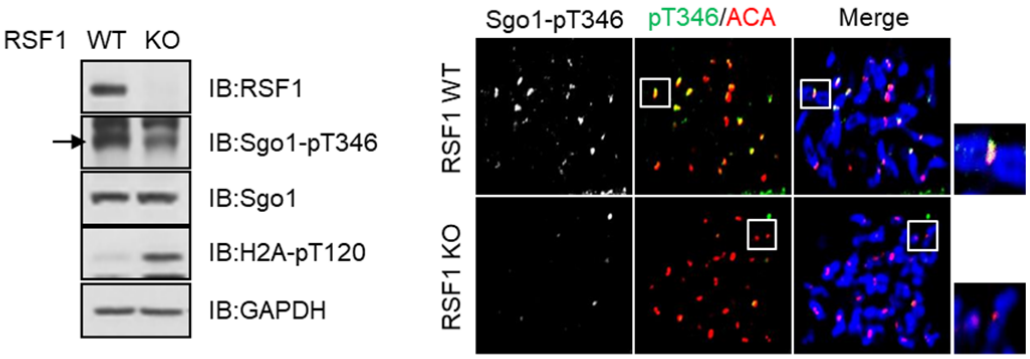

- Lines 120-131, and 468: the authors mentioned H2A (K118R) and (K119R) a lot of times in the main text of their MS, but these mutants were never described in the Method section. Instead, the H2A mutants (K118A, K119A, K119Q) in the method section were never mentioned in the main text. Thus, the authors looked carefully through their MS to correct this issue.

→ In the revised manuscript, we added the description of these histone mutants on both Result (lines 124-125) and Methods (lines 447-452) sections.

Minor issues

- line 166, "Although the transcriptional function of HDAC1 has been studied extensively, its subcellular localization and function in mitosis remain unknown." Actually, a few papers had reported the related information. These publications should be cited and discussed. I listed a few publications at below.

Dynamic phosphorylation of Histone Deacetylase 1 by Aurora kinases during mitosis regulates zebrafish embryos development. Loponte S, et al., and Chiocca S. Sci Rep. 2016 Jul 26;6:30213.

Dynamic distribution of HDAC1 and HDAC2 during mitosis: association with F-actin. He S, Khan DH, Winter S, Seiser C, Davie JR. J Cell Physiol. 2013 Jul;228(7):1525-35.

HDAC1 inactivation induces mitotic defect and caspase-independent autophagic cell death in liver cancer. Xie HJ, Noh JH, Kim JK, Jung KH, Eun JW, Bae HJ, Kim MG, Chang YG, Lee JY, Park H, Nam SW. PLoS One. 2012;7(4):e34265.

MBD3 and HDAC1, two components of the NuRD complex, are localized at Aurora-A-positive centrosomes in M phase. Sakai H, Urano T, Ookata K, Kim MH, Hirai Y, Saito M, Nojima Y, Ishikawa F. J Biol Chem. 2002 Dec 13;277(50):48714-23.

→ In the revised manuscript, we changed it to '…. its subcellular localization and function in mitosis was only reported partly[37,38]' with two references suggested by the reviewer.

- Lines 217-233, the authors mentioned the GFP-CENP-B-HDAC1 construct, but they did not elaborate how they made the construct in the method section.

→ We added it to the Method section (lines 460-463).

REVIEWERS' COMMENTS:

Reviewer #1 (Remarks to the Author):

I found that authors did substantial revision and the paper was much improved. Images for sister chromatid separation are now much clearer. I also feel that authors also addressed concerns from all reviewers.

Reviewer #2 (Remarks to the Author):

Re-Review of the Nature Communications manuscript NCOMMS-18-07011-T by Ho-Soo Lee et al., entitled "The chromatin remodeler RSF1 controls centromeric histone modifications to coordinate chromosome segregation"

This reviewer had 4 major points of criticism towards the original manuscript. In their revised manuscript the authors have adequately addressed points 1) and 3). The problems with the other two points are based on misunderstandings, which I will try to explain below:

Ad 2)
I had written: "RSF1 depletion not only abolishes the enrichment of HDAC1, H2A T120 phosphorylation and Sgo1 at kinetochores/centromeres; at the same time all three concentrate at chromosome arms to an extent that exceeds their amount at centromeres under normal conditions. This hyper-accumulation cannot simply be explained by the sole block of centromeric localization. Could it be that RSF1 somehow actively suppresses HDAC1 binding to chromosome arms? The fact that in the absence of RSF1 H2A K118 acetylation is only found at centromeres and missing from arms would be consistent with this assumption. The authors should discuss this issue in more detail then they do so far."

Lee et al. responded: "...We think that HDAC1 and Sgo1 may bind the entire chromatin with low binding affinity but centromeric accumulation of these proteins is mediated by more specific regulation with high binding activity. Thus, loss of proper signaling necessary for centromeric accumulation result in the re-displacement of these proteins along chromosome arms, leading to hyper-accumulation at the chromatin level...."

If re-localization from centromeres to arms was all that is going on, then the total fluorescence signal on the chromosome should stay the same, which it doesn't. It appears that what arrives at chromosome arms is much more than just the displaced centromeric pool. I still think that "the authors should discuss this issue in more detail then they do so far".

Ad 4)
I had written: "As a rationale for the identification of a HDAC1-binding deficient RSF1 variant, it is stated that "the interaction of HDAC1 with its binding partners occurs through a consensus binding motif [LXCXE]. We searched for the LXCXE motif in RSF1 and...". This makes no sense! It has been reported (but also questioned by others) that HDAC1 interacts via a LxCxE-like motif with the pocket domain of Rb. Thus, one would have to look for a pocket domain within RSF1 (which is obviously not there) and not for a LxCxE-like motif because this is present in HDAC1. I therefore strongly suggest to remove the sentence: "These data demonstrate that RSF1 interacts with HDAC1 through an LXCXE motif,..."."

Lee et al. now write in their revised manuscript: "We searched for any putative binding motifs in the C-terminal region of RSF1 and found that LSSSE as a LXCXE-like motif is well conserved among higher vertebrates (Supplementary Fig. 3c). It is shown that the LXCXE motif within the pocket domain of RB is crucial for interacting with various cellular and viral proteins37."

This is still false!!! There is no LxCxE motif within the pocket domain of RB! Instead, the pocket domain binds to LxCxE motifs in other proteins! If HDAC1 contains a LXCXE-like motif, then RSF1 should have a pocket domain, which it does not.
I am not saying that the identified RSF1 variant is not compromised in HDAC1 binding; instead, I am saying that the rationale how it was found was plain wrong.

Minor point:
The authors should add the green circles resembling unmodified Thr120 to the first three nucleosomes shown left in figure 6g; otherwise they make the impression that TIP60 adds this residue to H2A....

A very important new issue is the question of the relative impact of acetylation versus malonylation of K118 of H2A. This issue arises from a recent publication in Ishiguro et al. in Scientific Reports (DOI:10.1038/s41598-018-26114-z). Here, the authors claim that in yeasts the malonylation mimicking Lys to Asp/Glu variants prevent Bub1-dependent phosphorylation of H2A while the acetylation mimicking Lys to Arg variant does not. Maybe the authors could compare corresponding variants in some of their assays. At the very least, the authors have to cite and discuss this recent publication.

Reviewer #3 (Remarks to the Author):

The authors have addressed the concerns of this reviewer. The reviewer therefore voted for acceptance for publication.

Response to the Reviewer 2 (comments from reviewers in **blue,** responses in **black**)**:**

Re-Review of the Nature Communications manuscript NCOMMS-18-07011-T by Ho-Soo Lee et al., entitled "The chromatin remodeler RSF1 controls centromeric histone modifications to coordinate chromosome segregation"

This reviewer had 4 major points of criticism towards the original manuscript. In their revised manuscript the authors have adequately addressed points 1) and 3). The problems with the other two points are based on misunderstandings, which I will try to explain below:

Ad 2)
I had written: "RSF1 depletion not only abolishes the enrichment of HDAC1, H2A T120 phosphorylation and Sgo1 at kinetochores/centromeres; at the same time all three concentrate at chromosome arms to an extent that exceeds their amount at centromeres under normal conditions. This hyper-accumulation cannot simply be explained by the sole block of centromeric localization. Could it be that RSF1 somehow actively suppresses HDAC1 binding to chromosome arms? The fact that in the absence of RSF1 H2A K118 acetylation is only found at centromeres and missing from arms would be consistent with this assumption. The authors should discuss this issue in more detail then they do so far."

Lee et al. responded: "...We think that HDAC1 and Sgo1 may bind the entire chromatin with low binding affinity but centromeric accumulation of these proteins is mediated by more specific regulation with high binding activity. Thus, loss of proper signaling necessary for centromeric accumulation result in the re-displacement of these proteins along chromosome arms, leading to hyper-accumulation at the chromatin level...."

If re-localization from centromeres to arms was all that is going on, then the total fluorescence signal on the chromosome should stay the same, which it doesn't. It appears that what arrives at chromosome arms is much more than just the displaced centromeric pool. I still think that "the authors should discuss this issue in more detail then they do so far".

→ We thank the reviewer for the constructive feedback and added the reviewer's input to the revised manuscript. "……. Thus, loss of proper signaling necessary for centromeric accumulation result in the re-displacement of these proteins to chromosome arms. Moreover, RSF1 may somehow suppress HDAC1 binding to chromosome arms in normal conditions and thus, loss of RSF1 leads to hyper-accumulation of HDAC1 at chromosome arms (Fig. 3b, Supplementary Fig. 3a). In these conditions H2A-K118 acetylation is absent at chromosome arms (Fig. 2g), which increases the chance for phosphorylation of H2A-T120 (Fig. 5c), leading to the hyper-accumulation of H2A-T120 and Sgo1 in RSF1 KO cells. (lines 304-310)

Ad 4)
I had written: "As a rationale for the identification of a HDAC1-binding deficient RSF1 variant, it is stated that "the interaction of HDAC1 with its binding partners occurs through a consensus binding motif [LXCXE]. We searched for the LXCXE motif in RSF1 and...". This makes no sense! It has been reported (but also questioned by others) that HDAC1 interacts via a LxCxE-like motif with the pocket domain of Rb. Thus, one would have to look for a pocket domain within RSF1 (which is obviously

not there) and not for a LxCxE-like motif because this is present in HDAC1. I therefore strongly suggest to remove the sentence: "These data demonstrate that RSF1 interacts with HDAC1 through an LXCXE motif,

Lee et al. now write in their revised manuscript: "We searched for any putative binding motifs in the C-terminal region of RSF1 and found that LSSSE as a LXCXE-like motif is well conserved among higher vertebrates (Supplementary Fig. 3c). It is shown that the LXCXE motif within the pocket domain of RB is crucial for interacting with various cellular and viral proteins37."

This is still false!!! There is no LxCxE motif within the pocket domain of RB! Instead, the pocket domain binds to LxCxE motifs in other proteins! If HDAC1 contains a LXCXE-like motif, then RSF1 should have a pocket domain, which it does not.
I am not saying that the identified RSF1 variant is not compromised in HDAC1 binding; instead, I am saying that the rationale how it was found was plain wrong.

→ We apologize for our misknowledge and removed the sentence of "It is shown that the LXCXE motif within the pocket domain of RB is crucial for interacting with various cellular and viral proteins" in the revised manuscript.

Minor point:
The authors should add the green circles resembling unmodified Thr120 to the first three nucleosomes shown left in figure 6g; otherwise they make the impression that TIP60 adds this residue to H2A....

→ We added the green circles for unmodified Thr120 to the first three nucleosomes in figure 6g.

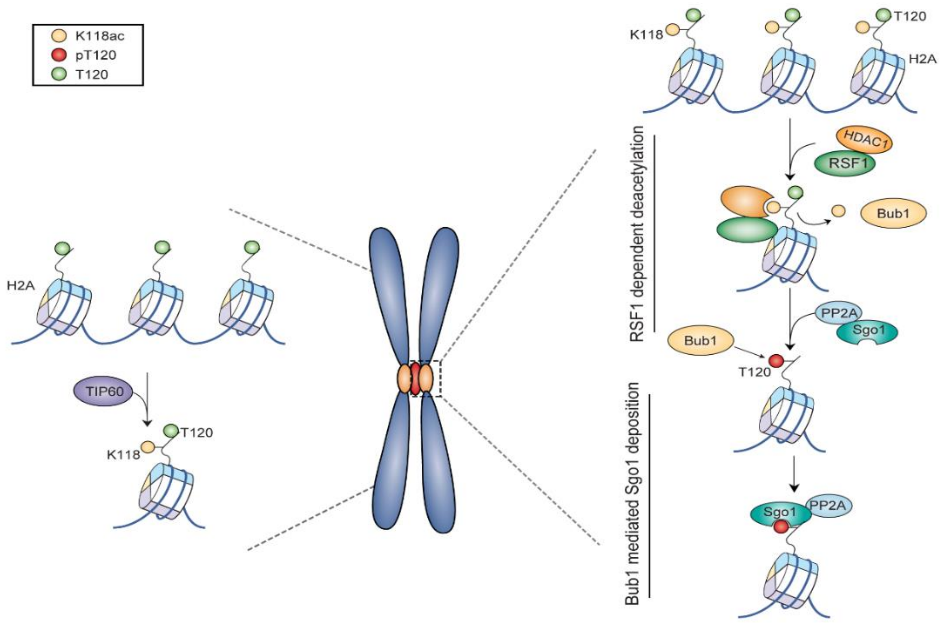

A very important new issue is the question of the relative impact of acetylation versus malonylation of K118 of H2A. This issue arises from a recent publication in Ishiguro et al. in Scientific Reports

(DOI:10.1038/s41598-018-26114-z). Here, the authors claim that in yeasts the malonylation mimicking Lys to Asp/Glu variants prevent Bub1-dependent phosphorylation of H2A while the acetylation mimicking Lys to Arg variant does not. Maybe the authors could compare corresponding variants in some of their assays. At the very least, the authors have to cite and discuss this recent publication.

→ We thank the reviewer for the comment. As the reviewer mentioned, the authors showed that in yeasts the malonylation of H2A prevented Bub1-dependent phosphorylation and Sgo1 accumulation. In the Fig. 2c and 2d of our manuscript, the acetylation mimicking H2A-K118Q (Lys to Gln) prevented Bub1-dependent phosphorylation while H2A-K118R (Lys to Arg) did not. Thus, histone modifications at the H2A-K118 would affect the Bub1-mediated phosphorylation in both humans and yeasts but they used different histone modifications.

We added the following sentences to the 'Discussion' section with reference (lines 285-289): 'Interestingly, a recent report showed that malonylation on H2A in yeasts prevented Bub1-mediated phosphorylation and Sgo1 accumulation[45]. Thus, histone modifications at the H2A-K118 would affect the Bub1-mediated phosphorylation in both humans and yeasts but they may utilize different histone modifications.'

45. Ishiguro T, Tanabe K, Kobayashi Y, Mizumoto S, Kanai M, Kawashima SA. Malonylation of histone H2A at lysine 119 inhibits Bub1-dependent H2A phosphorylation and chromosomal localization of shugoshin proteins. *Sci Rep* **8**, 7671 (2018).
